# Development and validation of the new HER2DX assay for predicting pathological response and survival outcome in early-stage HER2-positive breast cancer

**DOI:** 10.1016/j.ebiom.2021.103801

**Published:** 2022-01-03

**Authors:** Aleix Prat, Valentina Guarneri, Tomás Pascual, Fara Brasó-Maristany, Esther Sanfeliu, Laia Paré, Francesco Schettini, Débora Martínez, Pedro Jares, Gaia Griguolo, Maria Vittoria Dieci, Javier Cortés, Antonio Llombart-Cussac, Benedetta Conte, Mercedes Marín-Aguilera, Nuria Chic, Joan Anton Puig-Butillé, Antonio Martínez, Patricia Galván, Yi-Hsuan Tsai, Blanca González-Farré, Aurea Mira, Ana Vivancos, Patricia Villagrasa, Joel S. Parker, Pierfranco Conte, Charles M. Perou

**Affiliations:** a Translational Genomics and Targeted Therapies in Solid Tumors, August Pi i Sunyer Biomedical Research Institute (IDIBAPS), Barcelona, Spain; b Department of Medical Oncology, Hospital Clinic of Barcelona, Spain; c SOLTI cooperative group, Barcelona, Spain; d Department of Medicine, University of Barcelona, Barcelona, Spain; e Institute of Oncology (IOB)-Hospital Quirónsalud, Barcelona, Spain; f Department of Surgery, Oncology and Gastroenterology, University of Padova, Padova, Italy; Medical Oncology 2, Istituto Oncologico Veneto, IRCCS, Padova, Italy; g Department of Pathology, Hospital Clinic de Barcelona, Barcelona, Spain; h Reveal Genomics, Barcelona, Spain; i Molecular Biology CORE laboratory, Hospital Clinic de Barcelona, Barcelona, Spain; j Institute of Oncology (IOB)-Quiron, Madrid, Spain; k Vall d´Hebron Institute of Oncology (VHIO), Barcelona, Spain; l Department of Medical Oncology, Hospital Arnau de Vilanova, Valencia, Spain; m Biochemistry and Molecular Genetics Service, Hospital Clinic de Barcelona, Barcelona, Spain; n Centro de Diagnóstico Biomédico, Hospital Clinic, Barcelona, Spain; o Life Edit Therapeutics, North Carolina, USA; p Lineberger Comprehensive Cancer Center, University of North Carolina, Chapel Hill, USA

**Keywords:** HER2-positive breast cancer, HER2DX, Gene expression, Immune, Prognosis, Pathological complete response, Neoadjuvant, Risk of relapse, De-escalation

## Abstract

**Background:**

Both clinical and genomic data independently predict survival and treatment response in early-stage HER2-positive breast cancer. Here we present the development and validation of a new HER2DX risk score, and a new HER2DX pathological complete response (pCR) score, both based on a 27-gene expression plus clinical feature-based classifier.

**Methods:**

HER2DX is a supervised learning algorithm incorporating tumour size, nodal staging, and 4 gene expression signatures tracking immune infiltration, tumour cell proliferation, luminal differentiation, and the expression of the HER2 amplicon, into a single score. 434 HER2-positive tumours from the Short-HER trial were used to train a prognostic risk model; 268 cases from an independent cohort were used to verify the accuracy of the HER2DX risk score. In addition, 116 cases treated with neoadjuvant anti-HER2-based chemotherapy were used to train a predictive model of pathological complete response (pCR); two independent cohorts of 91 and 67 cases were used to verify the accuracy of the HER2DX pCR likelihood score. Five publicly available independent datasets with >1,000 patients with early-stage HER2-positive disease were also analysed.

**Findings:**

In Short-HER, HER2DX variables were associated with good risk outcomes (i.e., immune, and luminal) and poor risk outcomes (i.e., proliferation, and tumour and nodal staging). In an independent cohort, continuous HER2DX risk score was significantly associated with disease-free survival (DFS) (p=0·002); the 5-year DFS in the low-risk group was 97·4% (94·4-100·0%). For the neoadjuvant pCR predictor training cohort, HER2DX variables were associated with pCR (i.e., immune, proliferation and HER2 amplicon) and non-pCR (i.e., luminal, and tumour and nodal staging). In both independent test set cohorts, continuous HER2DX pCR likelihood score was significantly associated with pCR (p<0·0001). A weak negative correlation was found between the HER2DX risk score versus the pCR score (correlation coefficient -0·19).

**Interpretation:**

The two HER2DX tests provide accurate estimates of the risk of recurrence, and the likelihood to achieve a pCR, in early-stage HER2-positive breast cancer.

**Funding:**

This study received funding from Reveal Genomics, IDIBAPS and the University of Padova.

**Research in context**

**Evidence before this study**

We searched PubMed for clinical trials or studies published in English between Jan 1, 2010, and August 1, 2021, assessing HER2 inhibition in early-stage breast cancer, with the search terms “HER2-positive”, “early-stage”, “escalation”, “de-escalation”, “biomarker”, “breast cancer”, “tumour-infiltrating lymphocytes (TILs) in breast cancer”, and “anti-HER2 therapy”. In patients with early-stage HER2-positive breast cancer, clinical guidelines support the use of (neo)adjuvant anti-HER2-based targeting plus chemotherapy for most patients. However, various strategies to de-escalate systemic therapy have been evaluated, such as decreasing the amount of chemotherapy or the duration of trastuzumab. In addition, various strategies to escalate systemic therapy have also been explored, such as increasing HER2 blockade with either the addition of 1 year of pertuzumab to trastuzumab, or the addition of 1 year of neratinib after trastuzumab or switching to trastuzumab emtansine in patients who do not achieve a pCR following neoadjuvant trastuzumab-based chemotherapy. Despite the successes and limitations of these escalation and de-escalation strategies, most patients with early-stage, HER2-positive breast cancer are cured with chemotherapy and trastuzumab alone; therefore, there is a need for implementing new tools to help guide systemic therapies in early-stage, HER2-positive breast cancer, especially to identify those who do well when given the standard of care baseline therapy (i.e., trastuzumab and chemotherapy) and to identify those who need more, in light also of new promising drugs such as anti-HER2 antibody drug conjugates, and new tyrosine kinase inhibitors.

In 2020, we reported HER2DX, the first multivariable prognostic score in early-stage HER2-positive breast cancer that integrated tumour and nodal staging, TILs, intrinsic molecular subtype, and the expression of 13 individual genes. However, the first version of the HER2DX had three major limitations: 1) TILs in HER2DX are measured as a continuous variable (i.e., 0 to 100%) and the scoring of TILs suffers from difficulties of reproducibility across pathologists, 2) only 55 tumour genes were evaluated, and few were immune-related, and 3) HER2DX does not provide information regarding the likelihood of achieving a pathological complete response (pCR) following neoadjuvant anti-HER2-based therapy. This is important today as most patients with newly diagnosed stage 2-3 HER2-positive breast cancer are treated with neoadjuvant therapy.

**Added value of this study**

To our knowledge, the new HER2DX is the first assay that integrates clinical data with genomic data capturing tumour features, immune features, and pathology features all in one assay. In addition, HER2DX uses the information captured by the assay to predict two different clinical endpoints, namely, long-term survival outcome and likelihood of achieving a pCR. Interestingly, both HER2DX risk score and HER2DX pCR likelihood score provide complementary information, opening an opportunity to better guide therapy when used in combination.

**Implications of all the available evidence**

The evidence suggests that HER2DX risk score might be able to identify a substantial proportion of patients with early-stage, HER2-positive breast cancer who do not need additional therapies, such as pertuzumab, trastuzumab emtansine or neratinib, because of their favourable survival outcomes with chemotherapy and trastuzumab (plus endocrine therapy if hormone receptor-positive). Additional studies will further solidify the clinical utility of both HER2DX scores to help de-escalate systemic and/or loco-regional treatments, such as the duration of trastuzumab or the amount of chemotherapy.

Alt-text: Unlabelled box

## Introduction

HER2-positive breast cancer causes a substantial proportion of deaths.^1^ In the early stages, (neo)adjuvant chemotherapy plus trastuzumab (plus endocrine therapy in hormone receptor-positive disease) have consistently shown significant increases in survival.^2^ However, substantial clinical and biological heterogeneity exists in HER2-positive disease, which affects patients' prognosis and treatment benefit.^2^, ^3^, ^4^, ^5^

Strategies to either escalate or de-escalate systemic therapy in early-stage HER2-positive breast cancer to improve survival outcomes and quality of life have been explored,^6^ such as decreasing the number of cycles of chemotherapy and/or the duration of trastuzumab,^7^, ^8^, ^9^, ^10^ increasing HER2 blockade with pertuzumab^11^ or neratinib,^12^ or switching anti-HER2 therapy to trastuzumab emtansine in patients who do not achieve a pathological complete response (pCR) following neoadjuvant therapy.^13^ It is, however, clear that most patients with early-stage, HER2-positive breast cancer are cured with chemotherapy and trastuzumab alone.^2^ Therefore, the risk of overtreatment should be considered.

Several variables beyond tumour burden have been associated with patients´ prognosis and/or treatment response in early-stage, HER2-positive breast cancer. For example, the percentage of stromal tumour-infiltrating lymphocytes (TILs),^14^, ^15^, ^16^ hormone receptor status, and the intrinsic molecular subtypes of breast cancer^16^, ^17^, ^18^ are all linked to response and/or survival. However, decisions nowadays about escalation or de-escalation of systemic therapies are still based on traditional parameters, i.e., tumour size, nodal status, expression of the hormone receptors, and response to neoadjuvant therapy (i.e., pCR or not). Therefore, a tool that objectively integrates these multiple variables together will likely show better performance that any single feature, which would be a useful tool to help guide therapy in early-stage HER2- positive breast cancer.

In 2020, we reported HER2DX,^19^ a first attempt to build a multi-feature prognostic score in early-stage HER2-positive breast cancer. The score integrated information including tumour size and nodal staging, TILs, intrinsic molecular subtype, and the expression of 13 individual genes.^19^ However, TILs are measured as a continuous variable (i.e., 0 to 100%) and this scoring suffers from low rates of reproducibility across pathologists, even when cut-offs are used (kappa scores range 0.45-0.72).^20^ In addition, a limited set of 55 genes was evaluated, and few of these genes were immune-related. Finally, the first version of the HER2DX score was built to predict prognosis and did not provide specific information regarding the likelihood of achieving a pCR following neoadjuvant anti-HER2-based therapy. With these limitations in mind, here we describe the development and validation of a new HER2DX assay, a single 27-gene expression and clinical feature-based classifier able to provide two independent scores to predict both long-term prognosis and pCR likelihood in HER2-positive early breast cancer patients.

## Methods

### Study design and participants

**Figure 1:**
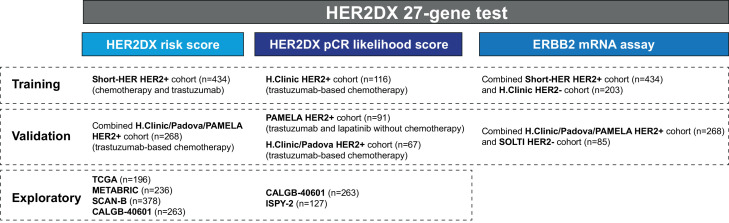
Summary of the different cohorts of patients evaluated during HER2DX development and validation.

A summary of all the cohorts evaluated is available in Figure. 1. Short-HER was a randomized, multicentric, investigator-driven phase 3 study, aimed to assess the non-inferiority of 9 weeks versus 1 year of adjuvant trastuzumab combined with chemotherapy.^7^ Briefly, women aged 18–75 with surgically resected, HER2-positive breast cancer, suitable for adjuvant chemotherapy were eligible. Women had to have node positivity, or in case of node-negativity, at least one of the following features: tumour size >2 cm, histological grade 3, presence of lympho-vascular invasion, Ki67 > 20%, age ≤35 years or hormone receptor negativity (i.e., oestrogen receptor and progesterone receptor <10%). Patients with stage IIIB/IV disease were not eligible. A total of 1,254 patients with a performance status of 0-1 were randomized from 17^th^ December 2007 to 6^th^ October 2013 to arm A or arm B. Chemotherapy in arm A (long) consisted of adriamycin 60 mg/m^2^ plus cyclophosphamide 600 mg/m^2^ or epirubicin 90 mg/m^2^ plus cyclophosphamide 600 mg/m^2^ every 3 weeks for 4 courses followed by paclitaxel 175 mg/m^2^ or docetaxel 100 mg/m^2^ every 3 weeks for 4 courses. Trastuzumab was administered every 3 weeks for 18 doses, starting with the first taxane dose. Chemotherapy in arm B (short) consisted of docetaxel 100 mg/m^2^ every 3 weeks for 3 courses followed by 5-fluorouracil 600 mg/m^2^, epirubicin 60 mg/m^2^, cyclophosphamide 600 mg/m^2^ every 3 weeks for 3 courses. Trastuzumab was administered weekly for 9 weeks, starting concomitantly with docetaxel. When indicated, radiation and hormonal therapy were carried out according to local standard. Median follow-up was 98·4 months.

SOLTI-1114 PAMELA was an open-label, single-group, phase 2 trial from 22^nd^ October 2013 to 30^th^ November 2015 aimed to the ability of the PAM50 HER2-enriched subtype to predict pCR at the time of surgery.^21^ Patients with HER2-positive disease, stage I–IIIA and a performance status of 0-1 were given lapatinib (1,000 mg per day) and trastuzumab for 18 weeks; hormone receptor-positive patients were additionally given letrozole (2.5 mg per day) or tamoxifen (20 mg per day) according to menopausal status. Treatment after surgery was left to treating physician discretion. Median follow-up was 68·1 months.

The Hospital Clinic and Padova University HER2-positive cohorts are consecutive series of patients with early-stage HER2-positive breast cancer and a performance status of 0-1 treated, as per standard practice, from 28th June 2005 to 26th September 2020 (Hospital Clinic) and 23rd February 2009 to 26th May 2016 (Padova University cohort), with neoadjuvant trastuzumab-based multi-agent chemotherapy for 3-6 months, followed by surgery. Adjuvant treatment was completed with trastuzumab for up to 1 year, and a minimum of 5 years of hormonal therapy for patients with hormone receptor-positive tumours. Radiation therapy was administered according to local guidelines. Median follow-up of Hospital Clinic and Padova University cohorts were 43·1 and 49·9 months, respectively.

Three publicly available gene expression-based datasets that included clinical data and survival outcome from patients with early-stage HER2-positive breast cancer treated with primary surgery were explored. All the data from The Cancer Genome Atlas (TCGA)^22^ and METABRIC^23^ datasets were obtained from the cbioportal webpage.^24^ The data from the SCAN-B dataset^25^ was obtained from GEO, under accession number GSE81540. The gene expression data from TCGA and SCAN-B is RNA-sequencing-based, whereas the gene expression data from METABRIC is microarray-based. No clear information regarding the type of locoregional and systemic therapy is available from these datasets, although patients in METABRIC did not receive anti-HER2 therapy.^23^

Two additional publicly available gene expression-based datasets that included clinical data and survival outcome from patients with early-stage HER2-positive breast cancer treated with neoadjuvant anti-HER2-based systemic therapy were also explored. The CALGB-40601 neoadjuvant study randomized 305 patients with stage II to III HER2-positive breast cancer to paclitaxel plus trastuzumab alone or with the addition of lapatinib for 16 weeks before surgery.^26^ An investigational arm of paclitaxel plus lapatinib (TL) was closed early. After surgery, it was recommended that all patients receive dose-dense doxorubicin and cyclophosphamide and complete 1 year of trastuzumab adjuvantly.^27^ The clinical data and the RNA-seq data of pre-treatment baseline samples from 263 of 305 (86·2%) patients from CALGB-40601 were downloaded from the dbGaP web site, under accession number phs001175. The second neoadjuvant public trial tested was the ISPY-2 study that adaptively randomized patients with clinical stage II to III HER2-positive breast cancer to T-DM1/pertuzumab, paclitaxel/trastuzumab/pertuzumab (THP), or a common control arm of paclitaxel/trastuzumab (TH), followed by doxorubicin/cyclophosphamide, then surgery.^28^ The microarray and clinical data from 127 of 128 (99·2%) patients in the ISPY-2 HER2-positive cohort was obtained from GEO, under accession number GSE181574.

Finally, we included a cohort of consecutive patients with newly diagnosed HER2-negative breast cancer screened for the SOLTI-1805 TOT-HER3 trial, a window-of-opportunity trial.^29^ Only baseline pre-treated tumours were analysed. No follow-up was available.

### Ethics

The study was performed in accordance with Good Clinical Practice guidelines and the World Medical Association Declaration of Helsinki. All patients provided informed consents. Approvals for the study were obtained from independent ethics committees.

### Tumour sample procedures

Gene expression assays were performed on tumour samples from Short-HER, TOT-HER3, PAMELA, and Padova University and Hospital Clinic of Barcelona cohorts at the Translational Genomics and Targeted Therapies in Solid Tumours at IDIBAPS. A minimum of ∼125 ng of total RNA was used to measure the expression of 185 breast cancer-related genes and 5 housekeeping genes (*GAPD, PUM1, ACTB, RPLP0 and PSMC4*) using the nCounter platform (Nanostring Technologies, Seattle, USA). The gene expression for each sample was independently normalized to the geometric mean of 5 housekeeping genes. Finally, TILs in Short-HER were assessed on a single haematoxylin–eosin stained.^30^ The data collected for the study cannot be made publicly available.

### HER2DX gene signatures

HER2DX is based on 4 different gene signatures comprising 27 genes, which capture various biological processes, including immune infiltration, tumour cell proliferation, luminal differentiation, and expression of the HER2 amplicon. The immune signature selected for HER2DX was the 14-gene immunoglobulin (IGG) module (i.e., *CD27, CD79A, HLA-C, IGJ, IGKC, IGL, IGLV3-25, IL2RG, CXCL8, LAX1, NTN3, PIM2, POU2AF1* and *TNFRSF17*), previously identified by unsupervised clustering of human breast tumours.^31^ The IGG signature has previously shown strong independent prognostic value in a large breast cancer dataset, where patients did not receive adjuvant systemic therapy.^31^ The other three gene signatures were identified from unsupervised clustering of the Short-HER HER2-positive dataset using data from 185-breast cancer-related genes (*data not shown*). The genes selected were obtained from highly correlated gene clusters (correlation coefficient > 0·80); the tumour cell proliferation signature includes 4 genes (i.e., *EXO1, ASPM, NEK2* and *KIF23*), the luminal differentiation signature includes 5 genes (i.e., *BCL2, DNAJC12, AGR3, AFF3* and *ESR1*), and the HER2 amplicon signature includes 4 genes located in the 17q11-12 chromosome (i.e., *ERBB2, GRB7, STARD3* and *TCAP*). For each signature, the normalized gene expression was calculated for each patient. Missing data was not imputed.

### Outcomes

The co-primary objectives of this study were to derive and validate two independently trained HER2DX scores: a prognostic risk score, and a pCR likelihood score. In the prognostic training dataset (i.e., Short-HER), the survival endpoint was distant relapse-free survival (DRFS), calculated as the time between randomization and distant recurrence or death before recurrence. In the validation prognostic dataset, the survival endpoint was disease-free survival (DFS) due to the availability of the data, which was calculated as the time between randomization and any of the following events, whichever first: local, regional, and distant recurrence; contralateral breast cancer, excluding in situ carcinoma; other second invasive primary cancer; death before recurrence or second primary cancer. In all neoadjuvant datasets, pCR at surgery was defined as no invasive tumour cells in the breast and axilla.

The secondary objectives were: 1) to describe the clinical-pathological features of the HER2DX risk groups; 2) to explore in-silico the association of HER2DX risk score with overall survival (OS) in publicly available datasets of HER2-positive early-stage breast cancer; 3) to evaluate the value of *ERBB2* mRNA to predict HER2 status according to the ASCO/CAP guidelines.^32^

### HER2DX risk score development and validation

The 434 patients enrolled in the Short-HER trial were used as the training dataset. Patient samples in the training dataset were split into a training set (67% of samples) and a testing set (remaining 33% of samples), balancing for DRFS event and treatment arm. Prognostic models of different feature sets were compared by C-index, the index of rank concordance for survival data, in the testing set. Tuning parameters in each feature set were evaluated by Monte-Carlo cross validation (MCCV) with 100 iterations. Cox proportional hazard models were fit with ridge regression or elastic net in each iteration of training and evaluated in the MCCV testing sets.

A single cut-off from the final HER2DX risk score was selected to split patients into low- and high-risk groups. The criteria to select this cut-off was that the low-risk group must have a lower boundary of the 95% confidence interval of the DRFS estimate above 90% at 3, 5 and 7 years. The final HER2DX risk score was tested, as a continuous variable and using the pre-specified cut-off, in 268 patients from the validation dataset. The validation dataset was composed of patients from Hospital Clinic of Barcelona HER2-positive cohort (n=147), PAMELA (n=84) and the Padova University cohort (n=37). The median follow-up of the validation dataset was 51·0 months.

To further evaluate the prognostic value of the HER2DX risk score, the HER2DX algorithm was evaluated in-silico across four publicly available datasets of patients with early-stage HER2-positive breast cancer (i.e., TCGA,^22^ METABRIC,^23^ SCAN-B^25^ and CALGB-40601^27^). HER2DX risk models with and without clinical variables (i.e., tumour and nodal staging) were explored as continuous variables due to the known technical biases between different genomic platforms.

### HER2DX pCR likelihood score development and validation

One-hundred and sixteen patients with early-stage HER2-positive breast cancer treated with neoadjuvant trastuzumab-based chemotherapy at Hospital Clinic of Barcelona were used as the training dataset for the HER2DX pCR likelihood score. Patient samples in the training dataset were split into a training set (67% of samples) and a testing set (remaining 33% of samples), balancing for pCR status. Logistic regression models were fit with ridge regression in each iteration of training and evaluated in the MCCV testing sets for parameters tuning. Two cut-offs based on tertiles in the training dataset were defined to split patients into three groups: low pCR likelihood, medium pCR likelihood and high pCR likelihood. The final HER2DX pCR likelihood score was tested, as a continuous variable and using the pre-specified cut-offs, in 158 patients from two validation datasets. The first validation dataset was composed of 67 patients treated with trastuzumab-based chemotherapy from Padova University cohort (n=37) and Hospital Clinic of Barcelona cohort (n=30). The second validation dataset was composed of 91 patients treated with neoadjuvant lapatinib and trastuzumab without chemotherapy from the PAMELA study.^21^ Finally, the HER2DX pCR likelihood score was evaluated in CALGB-40601^26^ and ISPY-2^28^ independent publicly available datasets.

### HER2DX ERBB2 mRNA expression assay

A cohort of 637 patients with primary invasive breast cancer and known HER2 status according to the ASCO/CAP guidelines^32^ was evaluated using the HER2DX assay and used as the training dataset to predict clinical HER2 status. This dataset was composed of 203 patients with newly diagnosed early-stage HER2-negative at Hospital Clinic breast cancer and the Short-HER HER2-positive cohort of 434 patients. The optimal cut-off of *ERBB2* expression to predict HER2 clinical status (positive versus negative) was obtained from a receiver operation curve and Youden index analysis. The optimal *ERBB2* cut-off was validated in an independent cohort of 353 HER2-negative and HER2-positive cases from the SOLTI-1805 TOT-HER3 HER2-negative trial (n=85), Hospital Clinic of Barcelona HER2-positive cohort (n=147), PAMELA (n=84) and Padova University cohort (n=37).

### General statistical procedures

For description purposes, 3-, 5- and 7-year estimates of DRFS or DFS were calculated by Kaplan-Meier. Univariate and multivariable Cox proportional hazard regression analyses were used to investigate the association of each variable with survival outcome. To evaluate the prognostic contribution of each variable, likelihood ratio values (χ2) were used to measure and compare the relative amount of prognostic information. Categorical variables were expressed as number (%) and compared by χ^2^ test or Fisher's exact test. Logistic regression analyses were performed to investigate the association of each variable with pCR. C-index and receiver operating characteristic (ROC) curves were used as a performance measure. The significance level was set to a 2-sided alpha of 0·05. We used R version 4.0.5. for all the statistical analyses.

### Role of the funding source

The study was designed and performed by investigators from Padova University, Hospital Clinic and Reveal Genomics. All authors had full access to all data in the study and had final responsibility for the decision to submit for publication.

## Results

### HER2DX risk score development and validation

To build a prognostic model, clinical-pathological and gene expression data were available from 434 (35%) of 1,254 patients in the Short-HER trial (Table 1**,** Fig. 1 and **Fig. S1-4**). Mean age was 55·4 (standard deviation [SD] 10·2) and most tumours were 2 cm or less (T1 stage), node-negative (N0 stage), hormone receptor-positive and histological grade 3. According to a modified version of Adjuvant! Online scoring system (version 8 with HER2 status),^33^ 376 of 429 (88·0%) patients had clinically high-risk disease. In this cohort, our previous study^19^ showed that the best prognostic models integrated tumour size, nodal status, TILs, and the main biology associated with the 4 intrinsic subtypes. Based on these previous findings, we re-develop HER2DX risk score based on 4 gene expression-based signatures tracking adaptive immune cell infiltration, tumour cell proliferation, HER2 amplicon expression and tumour cell luminal differentiation, together with tumour stage (T1 vs. T2 vs. T3-4) and nodal stage (N0 vs. N1 vs. N2-3). To capture immune infiltration, we selected our previously described IGG signature, which has shown a strong prognostic value in early-stage breast cancer.^31^ HER2DX variables were associated with good outcomes (i.e., immune/IGG, and luminal) and poor outcomes (i.e., proliferation, and tumour and nodal staging) when tested in univariate analyses. HER2 amplicon signature was not significantly associated with outcome. Overall, the accuracy (C-index) of the HER2DX risk score in Short-HER was 0·74, which was very similar (0·72) to the C-index of our previously reported HER2DX risk model based on 17 different variables.^19^ Of note, when we tried to add more variables into the current HER2DX risk model, including TILs, intrinsic subtypes, and individual genes, the C-index HER2DX did not improve (*data not shown*).

**Table 1:** Patient baseline characteristics of the Short-HER dataset.

	All patients		HER2DX Low-Risk		HER2DX High-Risk		
	N	%	N	%	N	%	p-value*
**N**	434	-	216	49·8%	218	50·2%	-
**Mean age**	55·4	-	55·6		55·1	-	0·580
**TILs**							0·004
TILs 0-29	378	87·1%	178	82·4%	200	91·7%	
TILs ≥30	56	12·9%	38	17·6%	18	8·3%	
**pT**							<0·001
T1	234	53·9%	152	70·4%	82	37·6%	
T2	187	43·1%	63	29·2%	124	56·9%	
T3-4	13	3·0%	1	0·4%	12	5·5%	
**pN**							<0·001
N0	235	54·2%	208	96·3%	27	12·4%	
N1	134	30·8%	8	3·7%	126	57·8%	
N2-3	65	15·0%	0	0·0%	65	29·8%	
**Estrogen receptor status**							
Positive	321	74·0%	155	71·8%	166	76·1%	0·326
Negative	113	26·0%	61	28·2%	52	23·9%	
**Treatment arm**							
Arm A (long)	221	50·9%	112	51·2%	109	50·0%	0·702
Arm B (short)	213	49·1%	104	48·2%	109	50·0%	
**Grade**							0·334
Grade 1	6	1·4%	0	0·0%	6	2·8%	
Grade 2	115	26·8%	65	30·5%	50	23·1%	
Grade 3	308	71·8%	148	69·5%	160	74·1%	
**Intrinsic subtype**							0·008
Luminal A	128	29·5%	65	30·1%	63	28·9%	
Luminal B	36	8·3%	10	4·6%	26	11·9%	
HER2-enriched	213	49·1%	104	48·2%	109	50·0%	
Basal-like	25	5·7%	14	6·5%	11	5·0%	
Normal-like	32	7·4%	23	10·6%	9	4·1%	

TILs: tumour-infiltrating lymphocytes

⁎ p-values represent comparison between HERDX low-risk and high-risk groups using χ^2^ test.

**Figure 2:**
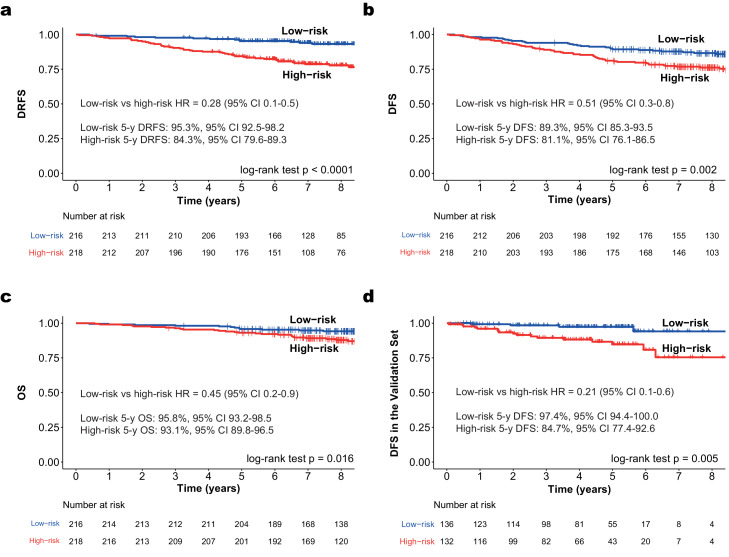
Survival outcomes of HER2DX low- and high-risk groups in early-stage HER2-positive breast cancer. (**a**) DRFS in Short-HER dataset (n=434); (**b**) DFS in Short-HER dataset (n=434); (**c**) OS in Short-HER dataset (n=434); (**d**) DFS in an independent combined validation dataset (n=268).

HER2DX risk score evaluated as a continuous variable was significantly associated with DRFS in the Short-HER 434 patient-dataset (p<0·0001; [cox-model]). To select a clinically relevant cut-off, we defined low-risk as a group of patients with a 3-, 5- and 7-year DRFS with a lower boundary of the 95% confidence interval (CI) >90%. This selected cut-off identified 49·8% of patients (n=216) as low risk. The 3-, 5- and 7-year DRFS of the low-risk population was 97·7% (95% CI 95·7-99·7), 95·3% (95% CI 92·5-98·2) and 94·3% (95% CI 91·2-97·4), respectively (Fig. 2**A**). The 3-, 5- and 7-year DRFS of the high-risk population was 90·4% (95% CI 86·5-94·4), 84·3% (95% CI 79·6-89·3) and 79·5% (95% CI 74·3-85·1), respectively. The DRFS, DFS and OS hazard ratios (HRs) between the low- and high-risk groups were 0·28 (95% CI 0·1-0·5), 0.51 (95% CI 0·3-0·8) and 0·45 (95% CI 0·2-0·9), respectively (Fig. 2**A-C**). In terms of clinical-pathological characteristics, the two risk-groups showed statistically significant differences in terms of TILs, nodal status, tumour size, and intrinsic subtype (Table 1). No significant differences between the two treatment arms (i.e., 9 weeks versus 1 year) were observed according to the two risk-groups, although the separation of the survival curves was visually apparent in the HER2DX high-risk group (**Fig. S5-7**).

A dataset of 268 patients with early-stage HER2-positive disease obtained from a combined cohort of three neoadjuvant studies was used for an independent validation of the HER2DX risk score (the score was determined using pre-treatment specimens before starting neoadjuvant therapy; Table 2). The validation dataset was composed of 147 patients from Hospital Clinic, 84 (56%) of 151 from PAMELA and 37 from the Padova University cohort (**Fig. S8**). All patients received chemotherapy and 1 year of trastuzumab; 84 (31%) of 268 patients received dual HER2 blockade with lapatinib and trastuzumab for 4·5 to 6·0 months, and 66 (25%) of 268 received four to six cycles of neoadjuvant pertuzumab. Despite heterogeneity in systemic therapies, there were no significant differences in DFS across the three cohorts, or between patients treated with trastuzumab-only versus dual HER2 blockade (*data not shown*).

**Table 2:** Patient baseline characteristics of the combined prognostic validation dataset.

	All patients		HER2DX Low Risk		HER2DX High Risk		
	N	%	N	%	N	%	p-value*
**N**	268	-	136	50·7%	132	49·3%	-
**Mean age**	56·3	-	56·2	-	56·3	-	0.980
TILs*							0·984
TILs 0-29	220	85·3%	112	84·8%	108	85·7%	
TILs ≥30	38	14·7%	20	15·2%	18	14·3%	
**Clinical tumour stage**							<0·001
T1	84	21·3%	61	45·0%	23	17·4%	
T2-4	184	78·7%	75	55·0%	109	82·6%	
**Clinical nodal stage**							<0·001
N0	162	55·4%	136	100·0%	26	20·0%	
N1-3	106	44·6%	0	0%	106	80·0%	
**Pathological response**							0·734
pCR	118	44·0%	58	42·6%	60	45·5%	
Residual disease	150	56·0%	78	57·4%	72	54·5%	
**Hormone receptor status**							0·027
Positive	171	63·8%	96	70·6%	75	56·8%	
Negative	97	36·2%	40	29·4%	57	43·2%	
**Intrinsic subtype**							0·003
Luminal A	43	19·1%	30	22·1%	13	9·8%	
Luminal B	30	12·4%	15	11·0%	15	11·4%	
HER2-enriched	158	51·7%	67	49·2%	91	69·0%	
Basal-like	16	7·9%	8	5·9%	8	6·0%	
Normal-like	21	9·0%	16	11·8%	5	3·8%	
**Study**							0·673
PAMELA	84	31·3%	46	33·8%	38	28·8%	
HOSPITAL CLINIC	147	54·9%	72	53·0%	75	56·8%	
PADOVA	37	13·8%	18	13·2%	19	14·4%	

TILs: tumour-infiltrating lymphocytes; pCR: pathological complete response.

⁎ TILs are missing in 10 cases; P-values represent comparison between HERDX low-risk and high-risk groups using χ^2^ test.

In the independent prognostic dataset, HER2DX score as a continuous variable was significantly associated with DFS (HR 1·03, 95% CI 1·0–1·1, p=0·002; [cox-model]). In this dataset, for every 10-unit increase (from 0 to 100) in HER2DX risk score, there was a 30% increase in the hazard for the event. According to the prespecified cut-offs, the HER2DX low-risk group had longer DFS than the high-risk (HR 0·21, 95% CI 0·1-0·6, p=0·005, [cox-model]) (Figure. 2**D**). 5-year DFS in the HER2DX low-risk and high-risk groups was 97·4% (95% CI 94·4–100·0) and 84·7% (77·4–92·6), respectively. 7-year DFS in the HER2DX low-risk and high-risk groups was 94·1% (95% CI 87·5–100·0) and 75·4% (62·6–91·0), respectively. The C-index of the HER2DX risk score was 0·73 for all patients on the independent test dataset.

To further explore the prognostic value of the HER2DX risk score in the adjuvant setting, we interrogated three publicly available breast cancer datasets (i.e., TCGA,^22^ METABRIC^23^ and SCAN-B^25^), which include clinical data, overall survival (OS) outcome and gene expression data for a total of 810 patients with early-stage HER2-positive breast cancer (**Table S1**). The HER2DX algorithm was applied in each dataset with and without clinical features (i.e., tumour and nodal staging) (Table 3). A statistically significant association between HER2DX risk score as a continuous variable and OS was observed across the tested public datasets.

**Table 3:** Association of the HER2DX risk score* with overall survival across three publicly available datasets.

	HR	95% CI	p-value*	χ2
**SCAN-B (n=378)**				
HER2DX risk score (GEP)	5·0	2·4-10·6	<0·001	18·7
HER2DX risk score (GEP+Clinical)	2·8	1·9-4·1	<0·001	31·9
**TCGA (n=196)**				
HER2DX risk score (GEP)	5·8	2·4-13·8	<0·001	15·6
HER2DX risk score (GEP+Clinical)	4·0	1·8-8·6	0·001	15·4
**METABRIC (n=236)**				
HER2DX risk score (GEP)	2·2	1·2-3·7	0·007	7·31
HER2DX risk score (GEP+Clinical)	1·7	1·3-2·1	<0·001	22·0

⁎ HER2DX risk score was evaluated using the 4 gene expression-based variables (GEP), and the full HER2DX risk score which includes tumour and nodal staging (GEP+Clinical). To evaluate the prognostic contribution of each score, likelihood ratio values (χ2) were used to measure and compare the relative amount of prognostic information. HR, hazard ratio; CI, confidence interval. SCAN-B dataset (source: GSE81540); The Cancer Genome Atlas (TCGA) dataset (source: https://www.cbioportal.org/); METABRIC dataset (source: https://www.cbioportal.org/). P-values were obtained from a cox-model.

To further explore the prognostic value of the HER2DX risk score when patients are treated with neoadjuvant and adjuvant anti-HER2-based therapy, we interrogated the CALGB-40601 publicly available breast cancer dataset,^27^ which include clinical data, relapse-free survival, and overall survival. The HER2DX algorithm was applied without clinical features (i.e., tumour and nodal staging). A statistically significant association between HER2DX risk score as a continuous variable and RFS and OS was observed independently of treatment arm and pCR status (**Table S2** and **Fig. S9-10**).

### HER2DX risk score in small tumours

Patients with small HER2-positive breast cancers (i.e., T1N0 and T2N0 with a tumour size ≤ 3.0 cm) have generally very good prognosis (i.e., DFS > 90% at 7-years) when treated with adjuvant paclitaxel and trastuzumab.^34^ To evaluate the prognostic value of HER2DX risk score in patients with low tumour burden, we identified 191 and 82 patients with T1N0 or T2N0 (tumour size ≤ 3.0 cm) in SCAN-B^25^ and METABRIC^23^ datasets, respectively (**Table S4**). Compared to the APT trial, SCAN-B and METABRIC datasets combined had lower proportion of T1mic/a/b tumours (i.e., 15.3% versus 49.4%, p<0.001, χ^2^ test). When HER2DX risk score was evaluated as a continuous variable, a statistically significant association with RFS and OS was observed in METABRIC, and with OS in SCAN-B (**Fig. S11-13**). Of note, tumour stage (T1 vs T2) was not found significantly associated with survival outcome in both datasets (data not shown). Overall, these in-silico results support the strong prognostic value of HER2DX, even in small tumours.

### HER2DX pCR likelihood score development and validation

To build a predictive model, we evaluated the HER2DX assay in pre-treated tumours from 116 patients with early-stage HER2-positive breast cancer treated with neoadjuvant trastuzumab-based chemotherapy (Figure. 1 and Table 4). Mean age was 57.3 (SD 15·1) and most tumours were larger than 2 cm (T2-4 stage), node-negative (N0 stage), hormone receptor-positive and histological grade 3. The 4 gene signatures (i.e., HER2 amplicon, immune/IGG, luminal and proliferation) and the 2 clinical variables (i.e., tumour and nodal staging) were used to train a HER2DX pCR likelihood score. HER2DX variables were associated with pCR (i.e., immune/IGG, and proliferation) and non-pCR (i.e., luminal, and tumour and nodal staging). Overall, the predictive performance (AUC) of the HER2DX pCR likelihood score in the training dataset was 0·81.

**Table 4:** Patient characteristics of the training and validation neoadjuvant datasets.

	Validation cohorts					
	Training cohort		PAMELA		Clinic / Padova	
	**N**	**%**	**N**	**%**	**N**	**%**
**N**	116	-	91	-	67	-
**Chemotherapy backbone**	116	100%	0	0%	67	100%
**Anti-HER2 therapy**						
Trastuzumab-only	69	59·5%	0	0·0%	48	71·6%
Trastuzumab and lapatinib	0	0·0%	91	100·0%	0	0·0%
Trastuzumab and pertuzumab	47	40·5%	0	0·0%	19	28·4%
**Mean age**	57·3		56·0		56·2	
TILs*						
TILs 0-29	98	86·0%	75	82·4%	52	88·1%
TILs ≥30	16	14·0%	16	17·6%	7	11·9%
**Clinical tumour stage**						
T1	32	27·6%	36	39·6%	17	25·4%
T2-4	84	72·4%	55	60·4%	50	74·6%
**Clinical nodal stage**						
N0	65	56·0%	54	59·3%	45	67·2%
N1-3	51	44·0%	37	40·7%	22	32·8%
**Pathological response**						
pCR	60	51·7%	32	35·2%	30	44·8%
Residual disease	56	48·3%	59	64·8%	37	55·2%
**Hormone receptor status**						
Positive	79	68·1%	49	53·8%	48	71·6%
Negative	37	31·9%	42	46·2%	19	28·4%
**Intrinsic subtype**						
Luminal A	24	20·7%	10	11·0%	9	13·4%
Luminal B	10	8·6%	8	8·8%	13	19·4%
HER2-enriched	66	56·9%	62	68·1%	35	52·2%
Basal-like	8	6·9%	6	6·6%	2	3·0%
Normal-like	8	6·9%	5	5·5%	8	12·0%

TILs: tumour-infiltrating lymphocytes; pCR: pathological complete response.

⁎ TILs data is missing in 2 cases.

Two cohorts of 91 and 67 patients with early-stage HER2-positive disease treated with neoadjuvant anti-HER2-based therapy was used for an independent validation of the HER2DX pCR likelihood score (the score was determined at baseline before starting neoadjuvant therapy; Table 5). In both cohorts, HER2DX pCR likelihood score as a continuous variable was found statistically significantly associated with pCR (p<0·0001; [logistic regression]). Overall, the predictive performances (AUC) of the HER2DX pCR likelihood score in the PAMELA study and the trastuzumab-based chemotherapy cohort were 0·80 and 0·77, respectively. As expected, statistically significant differences in pCR rates across the three response groups (i.e., defined by tertiles, which were determined in the training dataset), were observed (Table 6).

**Table 5:** Patient characteristics of the training and validation neoadjuvant datasets combined according to HER2DX pCR likelihood score.

	HER2DX pCR likelihood score*						
	Low		Medium		High		
	N	%	N	%	N	%	*P*-value*
**N**	88	-	83	-	103	-	
**Chemotherapy backbone**	64	72·7%	58	69·9%	61	59·2%	0·110
**Anti-HER2 therapy**							
Trastuzumab-only	38	43·2%	39	47·0%	40	38·8%	0·249
Trastuzumab and lapatinib	24	27·3%	25	30·1%	42	40·8%	
Trastuzumab and pertuzumab	26	29·5%	19	22·9%	21	20·4%	
**Mean age**	56·5	-	53·2	-	58·2		
TILs*							
TILs 0-29	77	92·8%	73	90·1%	75	75·0%	0·001
TILs ≥30	6	7·2%	8	9·9%	25	25·9%	
**Clinical tumour stage**							
T1	21	23·9%	23	27·7%	41	39·8%	0·044
T2-4	67	76·1%	60	72·3%	62	60·2%	
**Clinical nodal stage**							
N0	57	64·8%	46	55·4%	61	59·2%	0·453
N1-3	31	35·2%	37	44·6%	42	40·8%	
**Hormone receptor status**							
Positive	82	93·2%	58	69·9%	36	35·0%	<0·001
Negative	6	6·8%	25	30·1%	67	65·0%	
**Intrinsic subtype**							
Luminal A	37	42·1%	5	6·0%	1	1·0%	<0·001
Luminal B	18	20·5%	10	12·1%	3	2·9%	
HER2-enriched	28	31·8%	56	67·5%	79	76·7%	
Basal-like	1	1·1%	1	1·2%	14	13·6%	
Normal-like	4	4·5%	11	13·2%	6	5·8%	

⁎ Groups using the pre-specified cut-offs are shown. TILs: tumour-infiltrating lymphocytes. TILs data is missing in 5 cases. P-values were obtained using χ^2^ test.

**Table 6:** pCR rates across the two validation neoadjuvant datasets according to HER2DX pCR likelihood score.

	Low		Medium		High		
	N	%	N	%	N	%	*P*-value*
pCR rates cohort 1*	6/26	23·1%	8/19	42·1%	16/22	72·7%	0·003
pCR rates cohort 2*	2/24	8·3%	4/25	16·0%	26/42	61·9%	<0·001

⁎ Validation cohort 1 includes 67 patients treated with trastuzumab-based chemotherapy. Validation cohort 2 includes 91 patients who participated in the PAMELA trial. Groups using the pre-specified cut-offs are shown. P-values were obtained using χ^2^ tests.

To further explore the predictive value of the HER2DX pCR likelihood score when patients are treated with neoadjuvant anti-HER2-based therapy, we interrogated the CALGB-40601 and ISPY-2 publicly available breast cancer datasets, which include gene expression data and pathological response data. The HER2DX algorithm was applied without clinical features (i.e., tumour and nodal staging) since either one of them is not available. In both datasets, a statistically significant association between HER2DX pCR likelihood score as a continuous variable and pCR was observed independently of treatment arm (**Table S5-6**). The predictive performance of the HER2DX pCR likelihood score in the both datasets was 0·80 (ISPY-2) and 0·71 (CALGB-40601).

### Relationships between both HER2DX scores

To determine the similarity (or lack thereof) between both HER2DX scores, we evaluated a combined HER2-positive dataset that included Short-HER (n=434) and the validation prognostic dataset (n=268). Overall, the correlation coefficient of both HER2DX scores was weak (i.e., -0·19). In patients with HER2DX low-risk prognostic score, 46·3% (163/352) were identified as HER2DX high likelihood of pCR and 53·7% (189/352) as HER2DX low/med likelihood of pCR. In patients with HER2DX high-risk prognostic score, 33·1% (116/350) were identified as having a HER2DX high likelihood of pCR and 66·9% (234/350) as having a HER2DX low/med likelihood of pCR.

### HER2DX ERBB2 mRNA expression assay

*ERBB2* mRNA expression within HER2-positive breast cancer can help identify patients with a high response to anti-HER2 therapies,^26^^,^^35^^,^^36^ including T-DM1.^37^^,^^38^ In addition, *ERBB2* mRNA expression can help identify HER2 status according to the ASCO/CAP guidelines.^25^ To build an *ERBB2* mRNA expression assay that tracks with clinical HER2 status, we combined the Short-HER HER2-positive cohort (n=434) with a HER2-negative cohort of patients newly diagnosed of early-stage breast cancer at Hospital Clinic (n=203) (Figure. 1). Overall, the mean ERBB2 expression (in log base 2) in HER2-negative and HER2-positive disease was -2·01 and 1·24, respectively (a 6·5-fold difference). The ROC AUC of *ERBB2* expression to predict clinical HER2 status was 0·97 with a 90% sensitivity and 98% specificity. Using Youden´s analysis, an optimal cut-off of -0·98 was identified; 3·4% of clinically defined HER2-negative cases were identified as *ERBB2*-positive by mRNA, and 9·7% of clinically defined HER2-positive cases were identified as *ERBB2*-negative/low.

The optimal cut-off to predict HER2 status was tested in an independent dataset of 85 HER2-negative and 268 HER2-positive cases (Figure. 1). Overall, the mean *ERBB2* expression (in log base 2) in HER2-negative and HER2-positive disease was -2·17 and 0·96, respectively (a 6·3-fold difference). The ROC AUC of *ERBB2* expression to predict clinical HER2 status was 0·96 with an 84% sensitivity and 100% specificity. No HER2-negative cases were identified as *ERBB2*-positive, and 16·4% of HER2-positive cases were identified as *ERBB2*-negative/low (**Table S7**).

## Discussion

**Figure 3:**
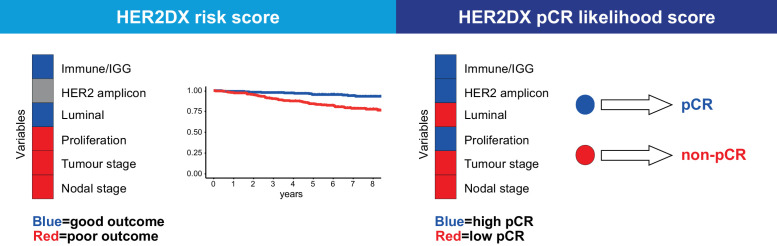
Summary of the variables included in the HER2DX assay and their association with each clinical endpoint. The type of association between a variable and each clinical endpoint is represented in different colours, where red means that a high score of that variable is associated with worse survival outcome or a lower likelihood of achieving a pCR, and blue means that a high score of that variable is associated with better survival outcome or a higher likelihood of achieving a pCR. Grey means no association of the variable with the clinical endpoint.

To our knowledge, this is the first study attempting to build a single assay that encompasses algorithms that provides independent prognostic and predictive information in early-stage HER2-positive breast cancer (Figure. 3). Specifically, HER2DX is associated with long-term survival and can identify groups of patients with large differences in their risk of relapsing following standard therapy (i.e., trastuzumab and chemotherapy). Additionally, HER2DX is associated with the likelihood to achieve a pCR if treated with anti-HER2-based therapy and can identify patients with large differences in their likelihood to achieve a pCR following neoadjuvant anti-HER2-based therapy. Interestingly, our study shows that each variable within HER2DX has a different relationship with therapy response in the neoadjuvant setting and long-term prognosis. This explains why the two HER2DX scores have a weak relationship among them. From a clinical point of view, HER2DX can identify patients with early-stage, HER2-positive disease who are candidates for escalated or de-escalated systemic treatment.

In stage 1 disease, 3-months of paclitaxel plus 1 year of trastuzumab is considered the standard of care for most patients based on the results of the APT trial,^34^ a single-arm study of 410 patients. Although this treatment strategy is now widely adopted, controversy exists in patients not entirely represented in the APT trial, such as those with hormone receptor-negative tumours or those with a tumour size between 2 to 3 cm. HER2DX could help better identify patients’ candidates for the APT treatment regimen. Regarding de-escalation of trastuzumab, several non-inferiority studies,^8^, ^9^, ^10^^,^^39^ including the Short-HER trial,^7^ have shown a small absolute reduction in risk of recurrence and a small absolute increase in risk of cardiac toxicity with 12 months of therapy compared with shorter durations. Although decreasing the duration of adjuvant trastuzumab has not been endorsed by clinical guidelines, HER2DX could help identify selected patients with low risk of recurrence, who would be ideal candidates for this treatment approach. For example, patients with important comorbidities or patients who experience cardiac toxicity and who have low HER2DX risk scores might be candidates for short duration trastuzumab treatments.

In stage 2-3 disease, escalated systemic treatments with pertuzumab, neratinib, and trastuzumab emtansine are being proposed during or after 1 year of trastuzumab. However, the absolute benefit of pertuzumab and neratinib is low (<3% in invasive disease-free survival). Trastuzumab emtansine, contrarily, has shown clinically meaningful results with an absolute increase in invasive disease-free survival at 3 years of 11.3% compared with trastuzumab in patients with HER2-positive breast cancer who do not achieve a pCR following standard anti-HER2-based chemotherapy. However, three of four patients in the control group of this pivotal trial did not have an event at 3 years. Moreover, several phase III trials are currently ongoing with new drugs in early-stage HER2-positive disease, such as tucatinib, abemaciclib, atezolizumab and trastuzumab deruxtecan. In this context, there is an urgent need to better define the population of patients with stage 2-3, HER2-positive disease who are candidates for escalated systemic therapies and avoid unnecessary toxicity and cost.

After a decade dissecting the molecular heterogeneity of HER2-positive breast cancer,^5^^,^^17^^,^^21^^,^^22^ we and others have elucidated the 4 main biological drivers of clinical behaviour, namely immune infiltration, luminal differentiation, tumour cell proliferation and HER2 amplicon expression. These biological drivers are captured by HER2DX and used in combination to predict two important clinical endpoints. Interestingly, immune infiltration/IGG^31^ is the only biological feature that is associated with a better response to neoadjuvant anti-HER2-based therapy and better survival outcome. Conversely, luminal differentiation and tumour cell proliferation have distinct associations with both clinical endpoints with luminal differentiation being associated with both a low response to neoadjuvant therapy and better survival outcome, and proliferation being associated with a higher response to neoadjuvant therapy but worse survival outcome. Finally, HER2 amplicon expression is not associated with survival outcome but shows an association with response to neoadjuvant anti-HER2-based therapy; these findings highlight the need for different algorithms, one to predict pCR and a second for survival, which HER2DX accomplishes. Similar results have been observed in the CALGB40601 HER2-positive neoadjuvant phase III trial,^27^ including the association of the IGG signature with more pCR and better survival outcome.

Apart from the main biological drivers, *ERBB2* mRNA levels by itself might provide useful clinical information. On one hand, we and others^25^^,^^40^^,^^41^ have shown the ability of *ERBB2* mRNA levels to predict clinical HER2 status according to the ASCO/CAP guidelines. On the other hand, *ERBB2* mRNA levels are associated with response to neoadjuvant anti-HER2-based therapy,^26^^,^^35^, ^36^, ^37^ including T-DM1^37^. In residual tumours following neoadjuvant anti-HER2-based chemotherapy, *ERBB2* mRNA was associated with T-DM1 survival benefit in the KATERINE phase III trial.^42^ Of note, *ERBB2* levels have shown to provide independent predictive information beyond the HER2-enriched molecular subtype.^35^ Finally, the field is moving away from a binary classification of HER2 (i.e., positive versus negative) and new entities are arising such as HER2-low disease,^43^^,^^44^ the latter of which is now being targeted by novel and potent anti-HER2 antibody drug-conjugates. Therefore, robust, and reproducible means of determining the levels of HER2 with a standardized assay with a larger dynamic range of HER2 expression by immunohistochemistry might become necessary soon.

Our study has some limitations. First, the validation prognostic dataset was a heterogeneous cohort of patients from three different sources. Second, a substantial proportion of patients in the validation prognostic dataset received trastuzumab in combination with lapatinib, which is not an approved anti-HER2 therapy in this setting. However, the absolute effect of 1-year of lapatinib when added to trastuzumab is known to be very small (i.e., 2% at 4 years).^45^ Third, the three patient cohorts from the validation prognostic dataset have different median follow-ups. Fourth, HER2DX risk score was developed from primary tumour specimens and staging was based on surgical pathology reports. This approach is different from the neoadjuvant setting where a core biopsy is the only available tissue and staging is based on imaging. Despite this limitation, HER2DX performed well in core biopsies in the validation prognostic dataset, where all patients received neoadjuvant therapy and clinical staging was used instead of pathology reports. Fifth, the Short-HER cohort was powered for a particular primary endpoint, which was to compare DFS between two arms distinguished by the duration of trastuzumab (i.e., 9 weeks versus 1 year).^7^ Here, due to the low sample size and number of events in each arm, we did not attempt to evaluate the value of HER2DX to predict the benefit from adjuvant trastuzumab according to its duration. Further retrospective and/or prospective analyses of HER2DX could explore this endpoint as well as other escalation or de-escalation treatment strategies.

To conclude, HER2DX is a novel 27-gene expression and clinical feature-based classifier intended for clinical use for patients with early-stage HER2-positive breast cancer. The assay integrates clinical data with genomic data capturing tumour- and immune-related biology and predicts two different clinical endpoints, namely, long-term survival and likelihood of achieving a pCR. We validate these two novel signatures, one for survival and one for predicting pCR, using multiple datasets, thus providing a high level of technical and clinical validation. Interestingly, the HER2DX risk score and HER2DX pCR likelihood score provide complementary information, opening an opportunity to better guide therapy through use of predictions of both response and survival.

## Data sharing statement

The data collected for the study cannot be made publicly available to allow for commercialization of research findings. However, we encourage investigators interested in data access and collaboration to contact the corresponding author (AP). The research-based R code to determine the HER2DX scores are available upon reasonable request to the corresponding author (AP).

## Contributors

AP, CMP and JSP designed the study. AP, PC, LP, YT, GG, FBM and TP contributed to data collection and assembly. AP, YT, LP, CMP and JSP interpreted and analysed the data. All authors wrote and reviewed the report and approved the final version for submission. AP, YT, FBM and LP verified the underlying data.

## Declaration of Competing Interest

Dr. Perou, Dr. Prat, Dr. Vivancos, Dr. Villagrasa, and Dr. Parker are equity stockholders of Reveal Genomics; Dr. Perou, Dr. Prat, Dr. Vivancos, and Dr. Parker are also consultants of Reveal Genomics. Dr. Prat reports grants from Reveal Genomics, during the conduct of the study; other from Reveal Genomics, personal fees from Roche, grants and personal fees from AstraZeneca, grants and personal fees from Daiichi-Sankyo, grants and personal fees from Novartis, personal fees from Foundation Medicine, personal fees from Oncolytics Biotech, outside the submitted work; In addition, Dr. Prat has a patent HER2DX licensed to Reveal Genomics, and a patent WO 2018/103834 licensed to Reveal Genomics. Dr. Paré has a patent HER2DX licensed to Reveal Genomics. Dr. Cortés reports grants and personal fees from Roche, personal fees from Celgene, personal fees from Cellestia, grants and personal fees from AstraZeneca, personal fees from Seattle Genetics, personal fees from Daiichi Sankyo, personal fees from Erytech, personal fees from Athenex, personal fees from Polyphor, personal fees from Lilly, personal fees from Merck Sharp&Dohme, personal fees from GSK, personal fees from Leuko, personal fees from Bioasis, personal fees from Clovis Oncology, personal fees from Boehringer Ingelheim, personal fees from Ellipses, personal fees from Hibercell, personal fees from BioInvent, personal fees from Gemoab, personal fees from Gilead, personal fees from Menarini, personal fees from Zymeworks, grants from Guardant health, grants from Pfizer, grants from Puma, non-financial support from Medsir, outside the submitted work; In addition, Dr. Cortés has a patent WO 2018/103834 licensed to Reveal Genomics. Dr. Dieci reports personal fees from Eli Lilly, MSD, Exact Sciences, Novartis, Pfizer, Seagen, outside the submitted work; In addition, Dr. Dieci has a patent HER2DX licensed to Reveal Genomics. Dr. Griguolo reports personal fees from Eli Lilly, Amgen, Novartis, Pfizer, Daiichi Sankyo, outside the submitted work; Dr. Guarneri reports personal fees from Eli Lilly, Roche, Novartis, MSD, GSK, Gilead, outside the submitted work; In addition, Dr. Guarneri has a patent HER2DX licensed to Reveal Genomics. Dr. Llombart-Cussac reports grants and personal fees from Roche, grants and personal fees from Daiichi Sankyo, personal fees from Pfizer, personal fees from Novartis, personal fees from Lilly, personal fees from MSD, personal fees from Agendia, from Exact Sciences, non-financial support from AstraZeneca, personal fees from Gilead, other from MedSir, outside the submitted work; In addition, Dr. Llombart-Cussac has a patent WO 2018/103834 licensed to Reveal Genomics. Dr. Villagrasa reports other from Reveal Genomics, personal fees from Nanostring, outside the submitted work; In addition, Dr. Villagrasa has a patent HER2DX pending. Dr. Conte reports personal fees from Roche, personal fees from Novartis, personal fees from Daiichi Sankyo, personal fees from Astrazeneca, personal fees from Elililly, outside the submitted work; In addition, Dr. Conte has a patent HER2DX pending. Dr. Brasó-Maristany has a patent New HER2DX assay licensed to Reveal Genomics. Dr. Vivancos reports personal fees from Bayer, personal fees from Bristol Meyers Squibb, personal fees from Guardant Health, personal fees from Merck, personal fees from Novartis, personal fees from Roche, personal fees from Incyte, outside the submitted work; In addition, Dr. Vivancos has a patent WO2015145388A3 licensed. Dr. Perou reports grants from Reveal Genomics, during the conduct of the study; other from Reveal Genomics, outside the submitted work. No authors have been paid to write this article.

## Appendix Supplementary materials

Image, application 1
