# Supplementary material for: Development and validation of the new HER2DX assay for predicting pathological response and survival outcome in early-stage HER2-positive breast cancer
Source: eBioMedicine. 2022 Jan 3;75:103801. doi: 10.1016/j.ebiom.2021.103801 (PMC8741424; doi:10.1016/j.ebiom.2021.103801)
Supplement: Supplementary file 1 [file mmc1.docx]

**Webappendix**

**Development and validation of the new HER2DX assay for predicting pathological response and survival outcome in early-stage HER2-positive breast cancer**

**Figure S1. Consort diagram of the Short-HER dataset.**

**Figure S2. Distant relapse-free survival (DRFS) outcomes of two treatment arms in early-stage HER2-positive breast cancer in the Short-HER trial.**

**
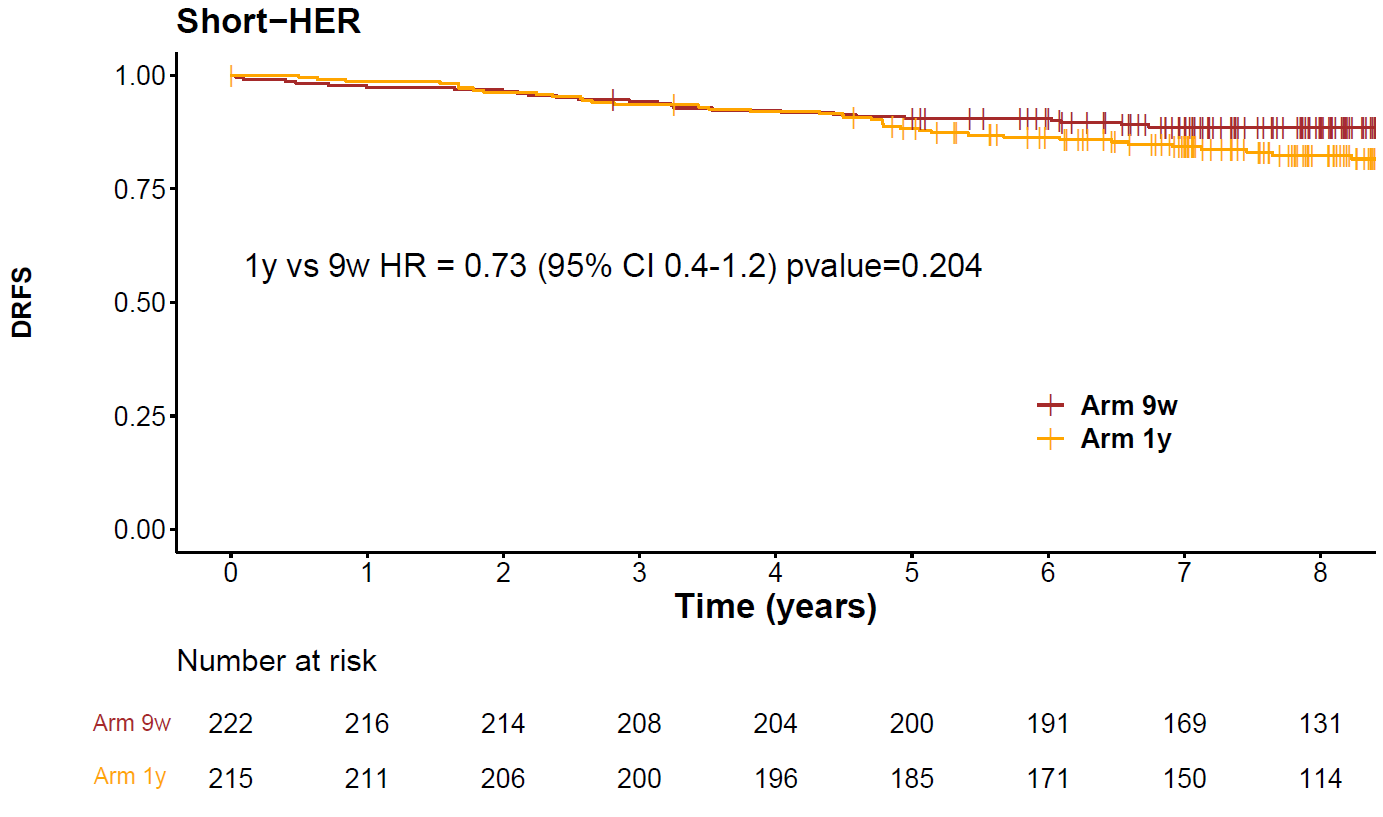
**

**Figure S3. Disease-free survival (DFS) of the two treatment arms in early-stage HER2-positive breast cancer in the Short-HER trial.**

**
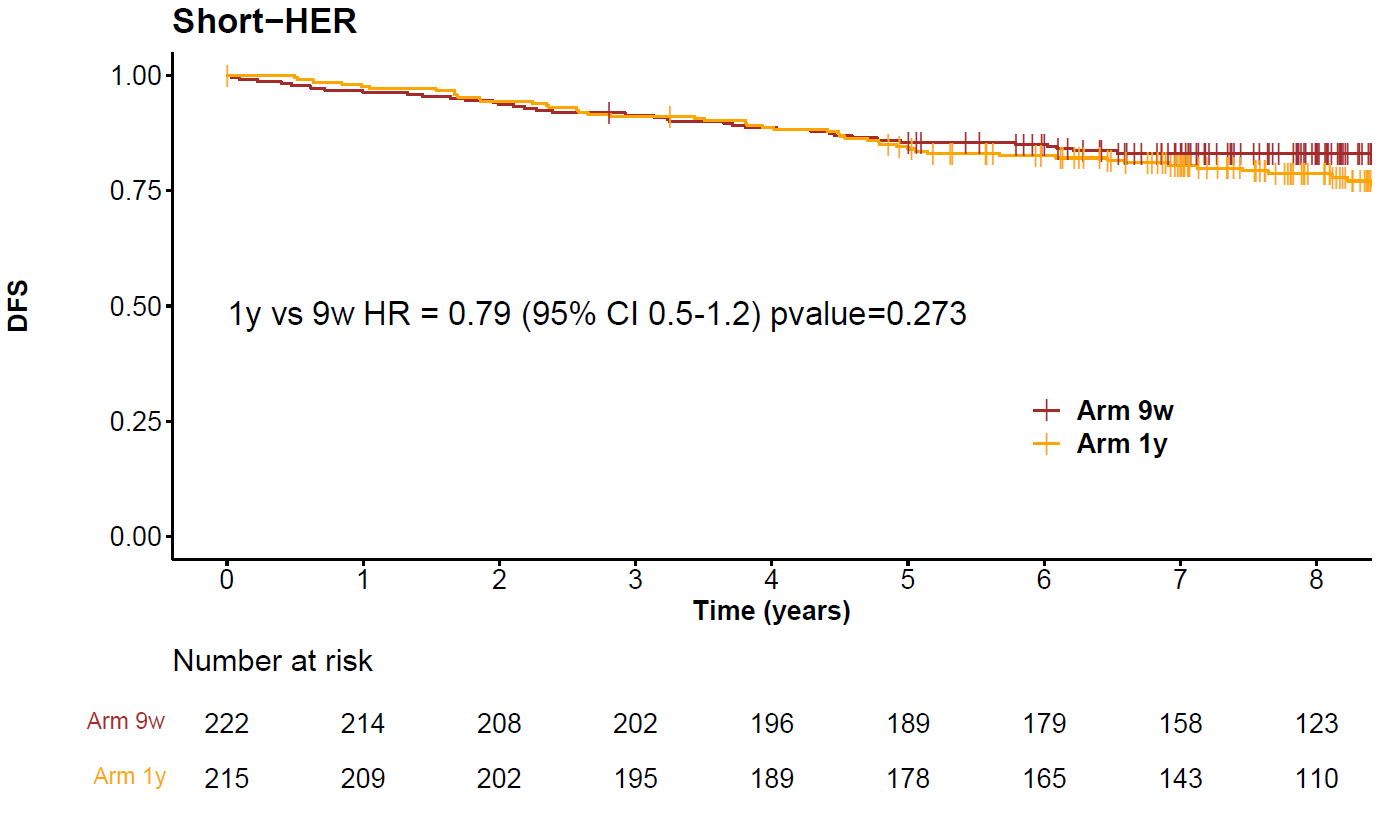
**

**
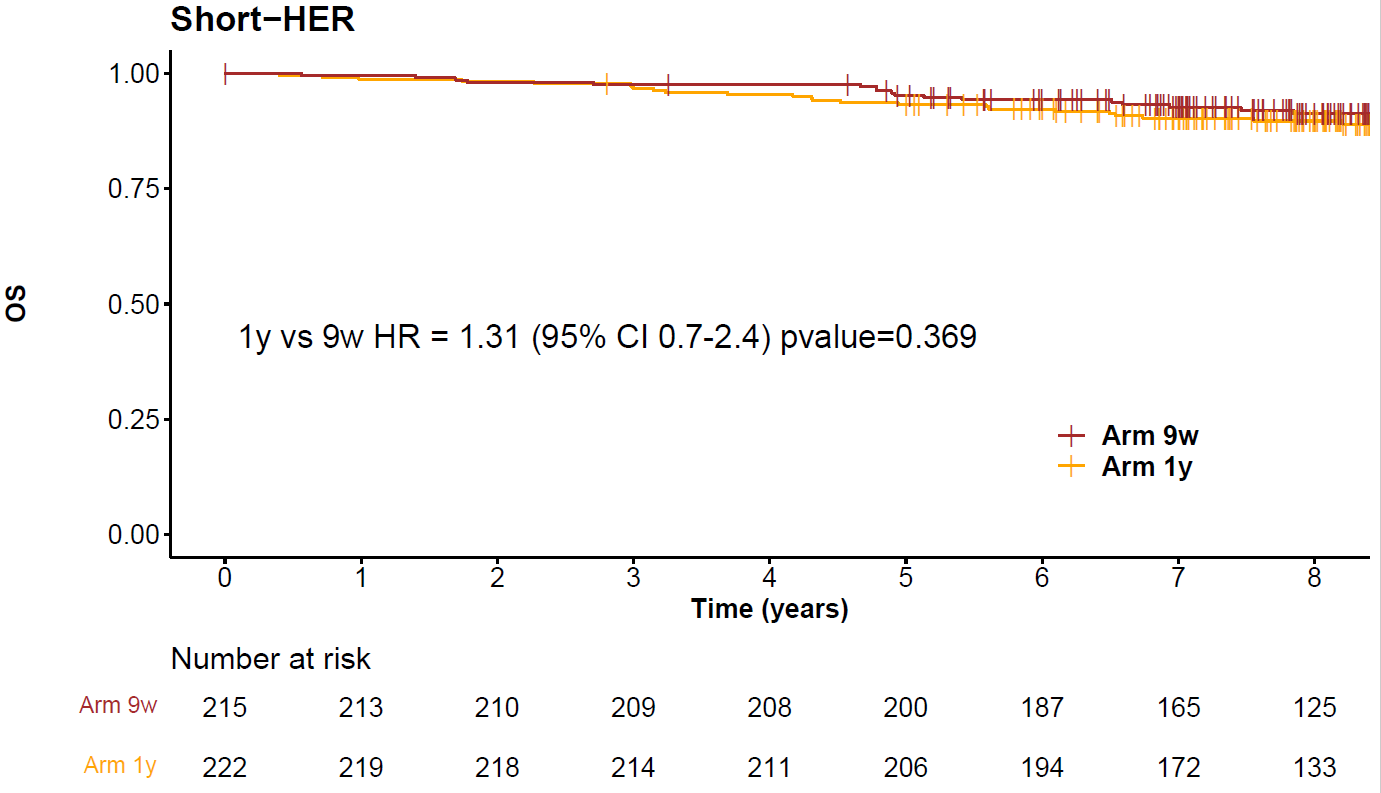
Figure S4. Overall survival (OS) outcomes of the two treatment arms in early-stage HER2-positive breast cancer in the Short-HER trial**

**
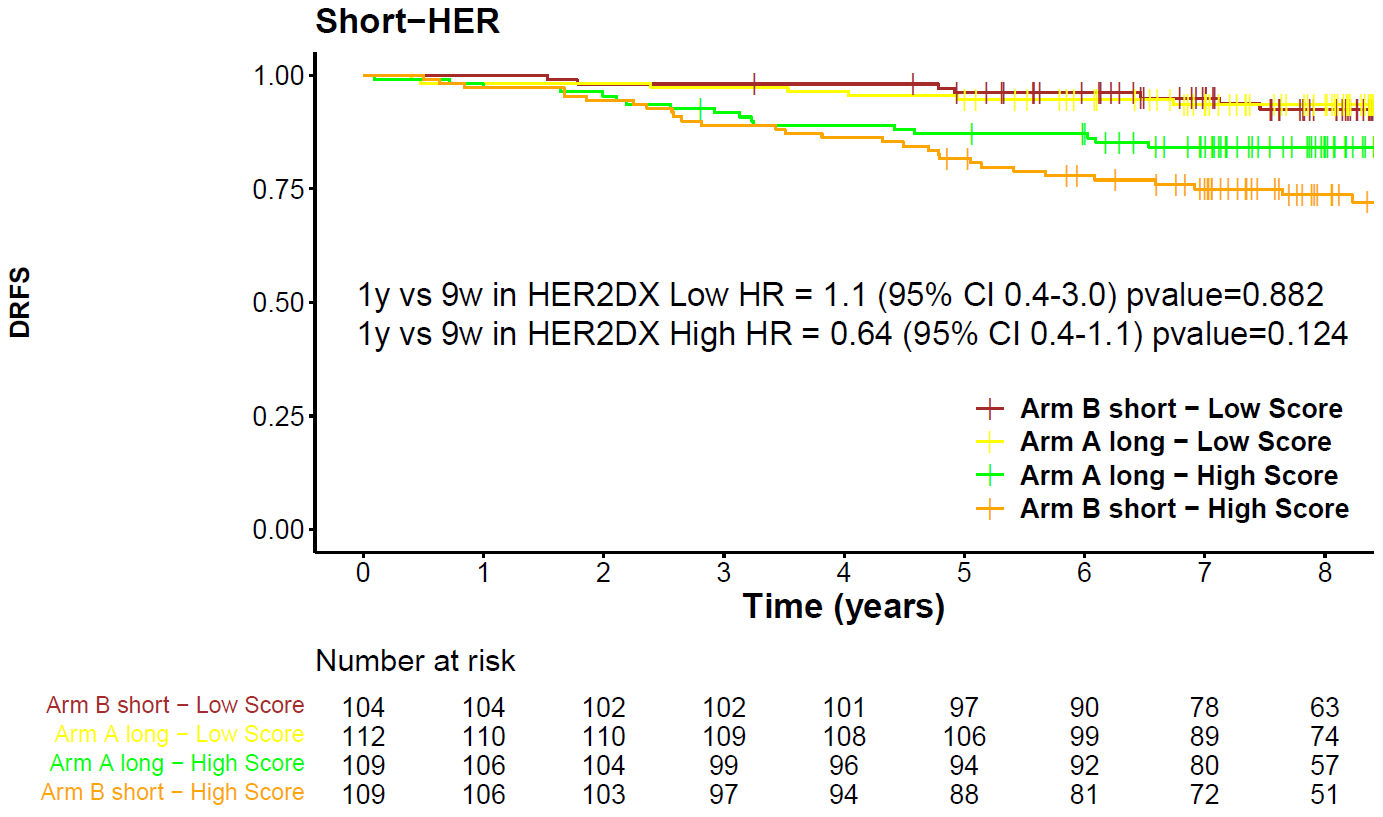
Figure S5. Distant relapse-free survival (DRFS) outcomes of HER2DX low- and high-risk groups in early-stage HER2-positive breast cancer in the Short-HER trial.**

**
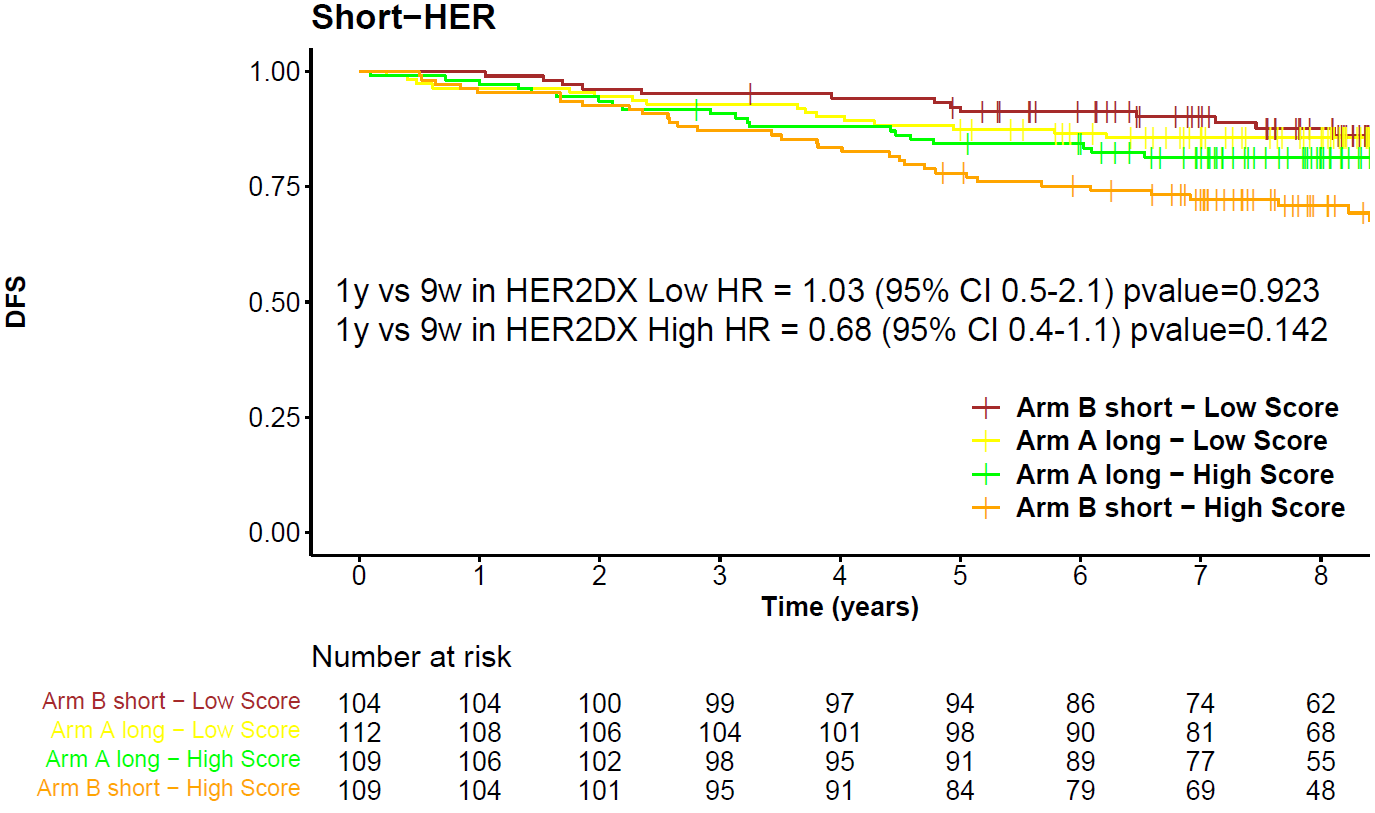
Figure S6. Disease-free survival (DFS) outcomes of HER2DX low- and high-risk groups in early-stage HER2-positive breast cancer in the Short-HER trial.**

**
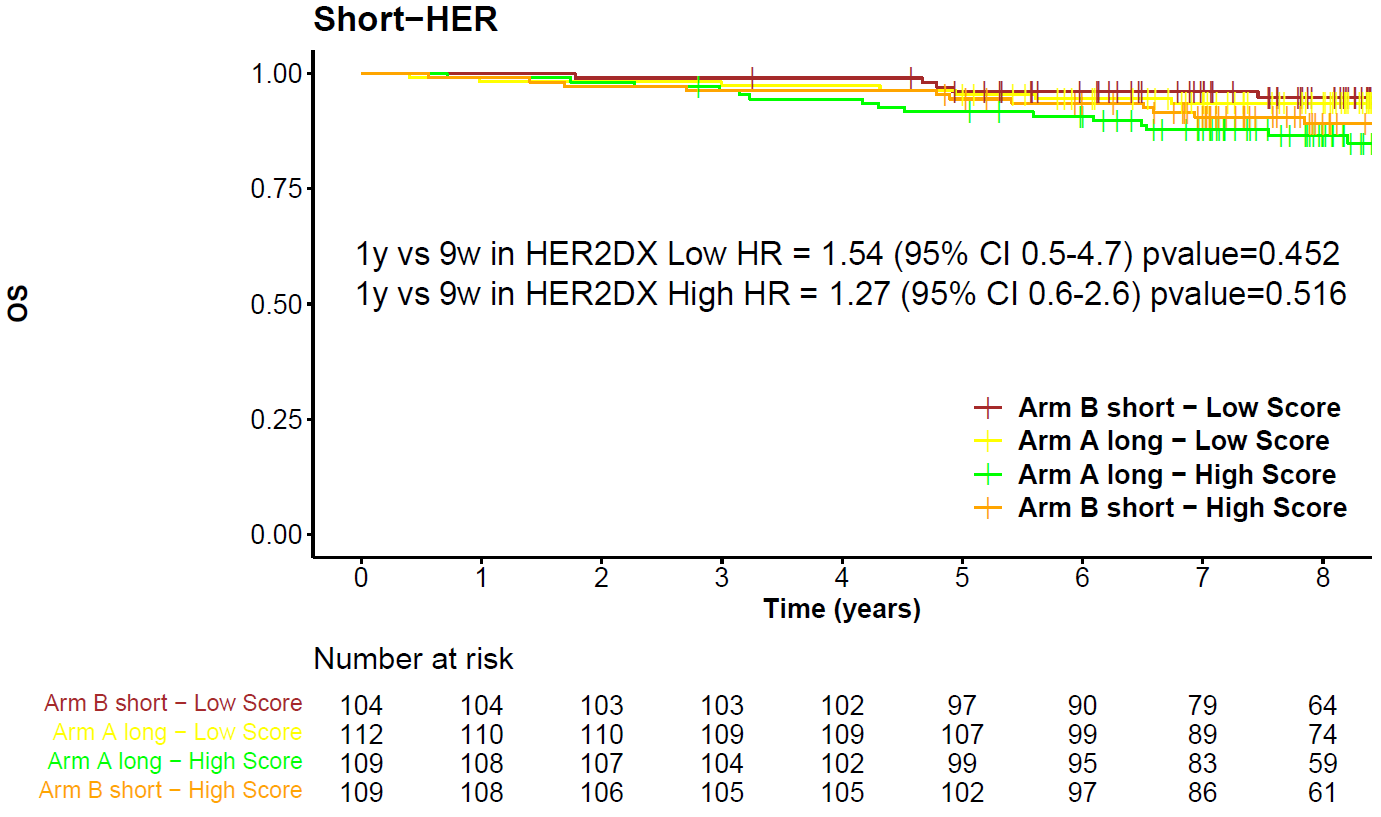
Figure S7. Overall survival (OS) outcomes of HER2DX low- and high-risk groups in early-stage HER2-positive breast cancer in the Short-HER trial**

**Figure S8. Consort diagram of the combined evaluation prognostic dataset.**

**Figure S9. Event-free survival (EFS) outcomes of HER2DX low- and high-risk groups in early-stage HER2-positive breast cancer in the CALGB40601 trial*.**

**
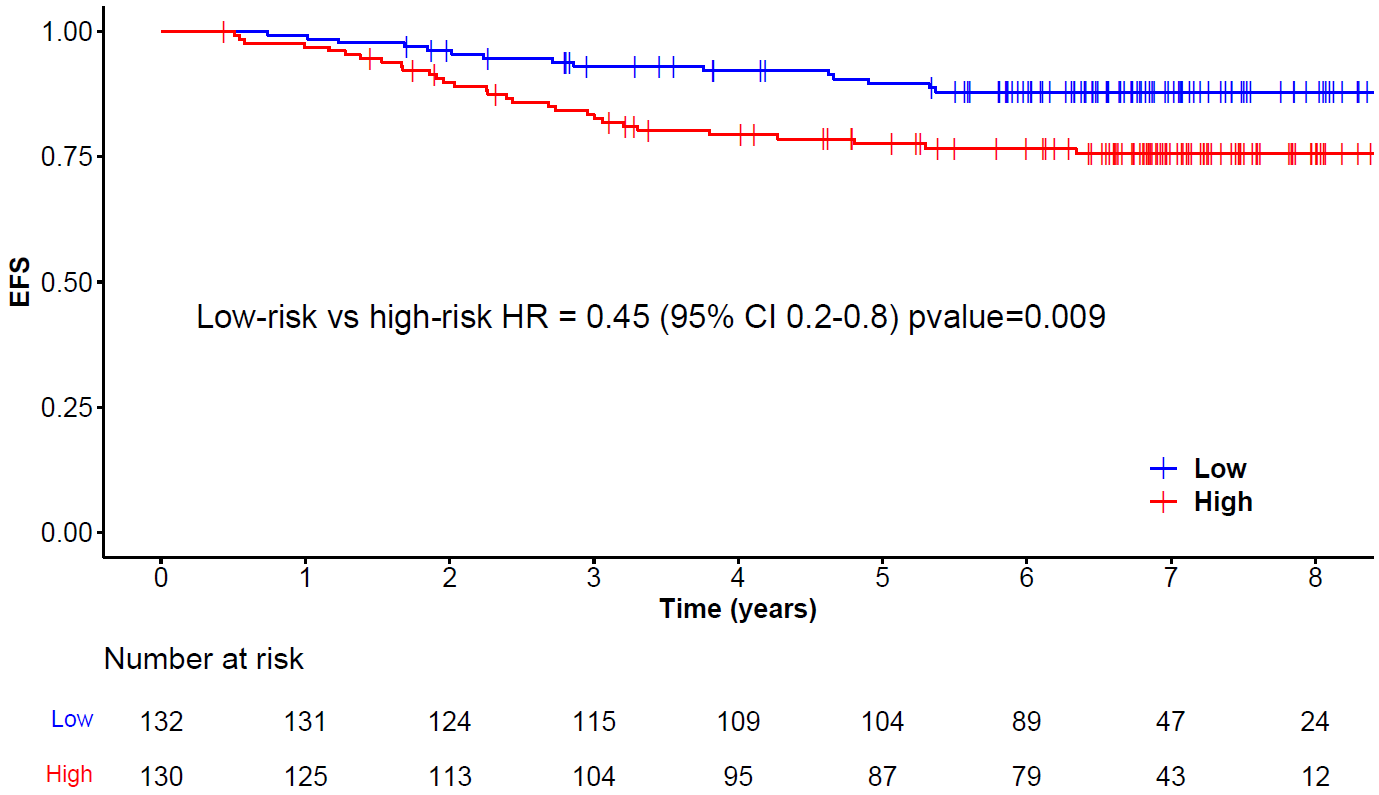
**

*HER2DX pCR score was obtained from RNA-seq-based publicly available data. Since clinical nodal status was not available, HER2DX risk score was calculated without clinical variables. Median cut-off was used to define low- and high-risk groups.

**Figure S10. Overall survival (OS) outcomes of HER2DX low- and high-risk groups in early-stage HER2-positive breast cancer in the CALGB40601 trial*.**

**
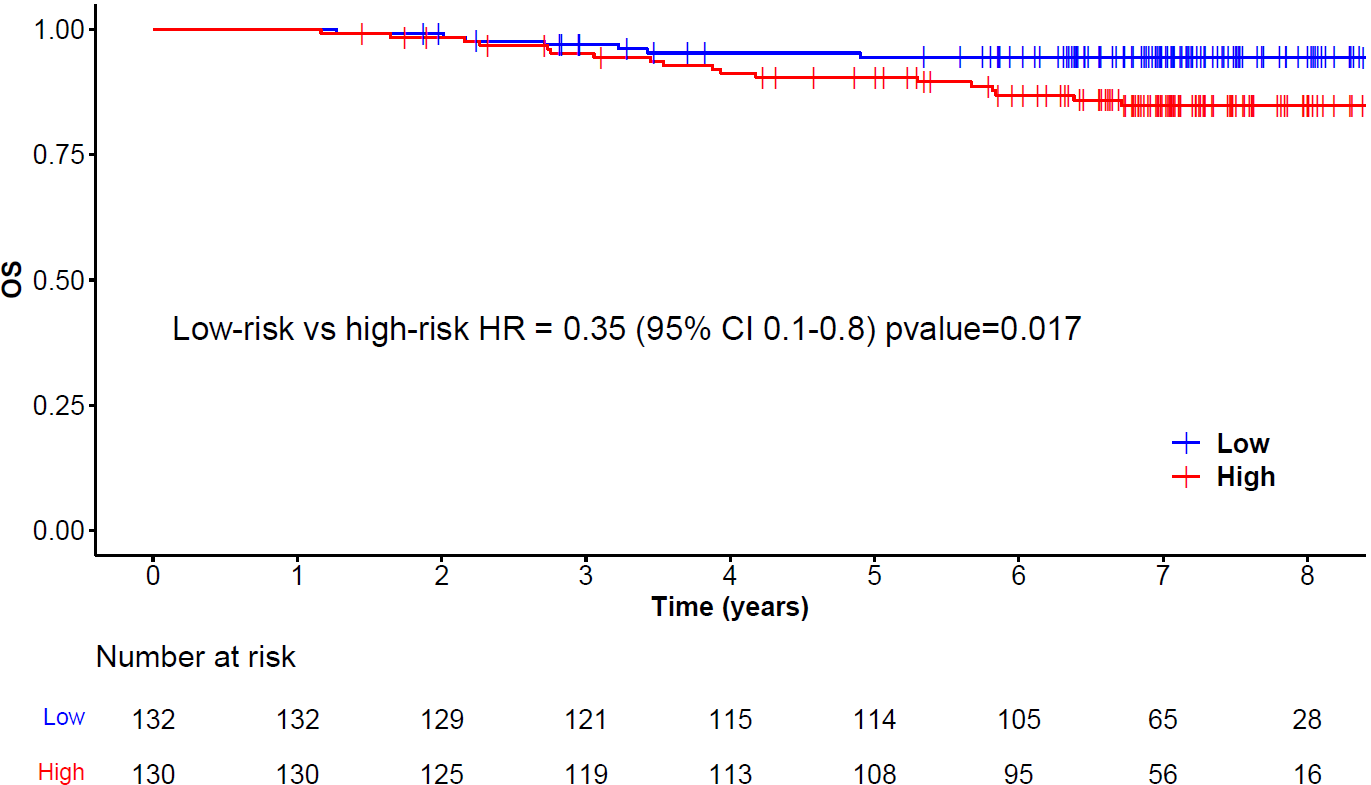
**

***** HER2DX pCR score was obtained from RNA-seq-based publicly available data. Since clinical nodal status was not available, HER2DX risk score was calculated without clinical variables. Median cut-off was used to define low- and high-risk groups.

**Figure S11. Relapse-free survival (RFS) outcomes of HER2DX risk score according to tertiles in T1N0 and T2N0 (tumour size ≤3cm) early-stage HER2-positive breast cancer in METABRIC*.**

**
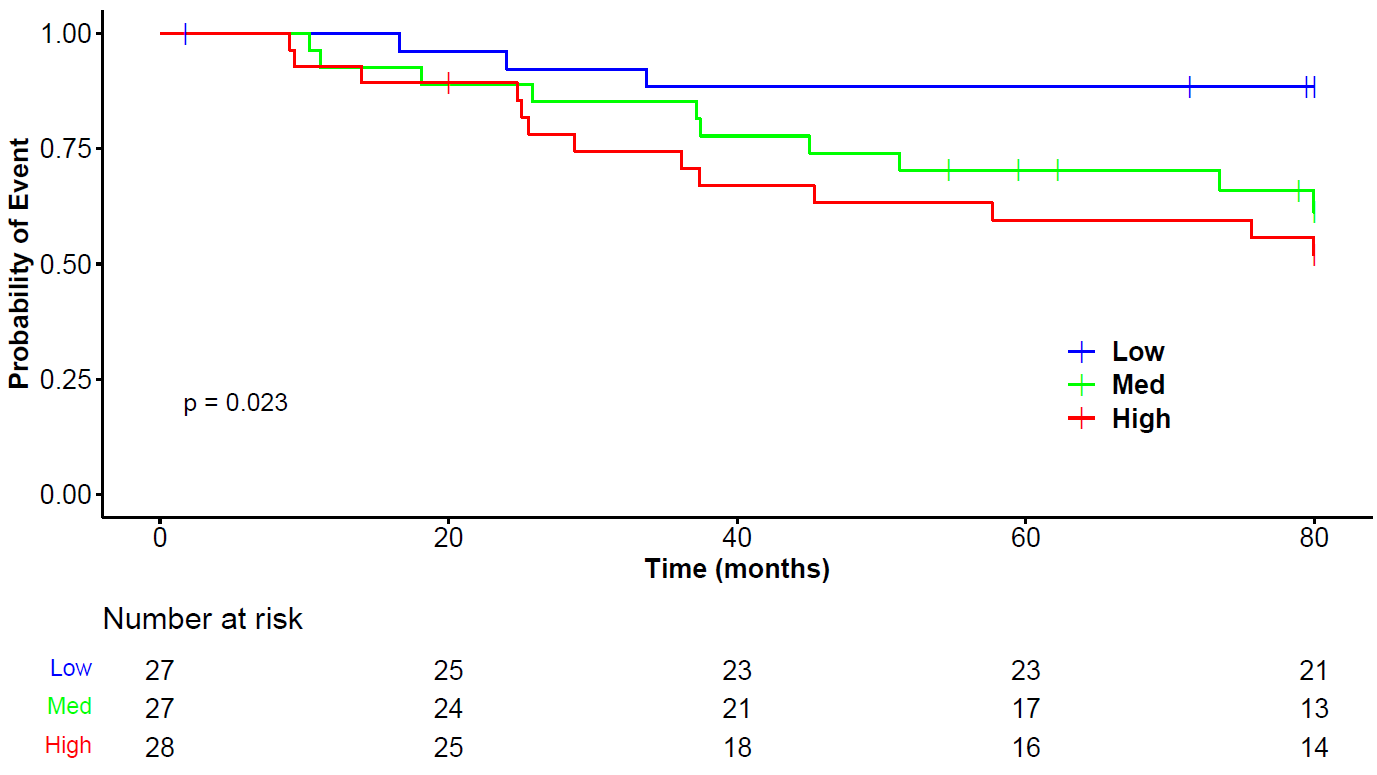
**

HER2DX pCR score was obtained from microaray-based publicly available data. Tertiles were used to define low-, medium- and high-risk groups

**Figure S12. Overall survival (OS) outcomes of HER2DX risk score according to tertiles in T1N0 and T2N0 (tumour size ≤3cm) early-stage HER2-positive breast cancer in SCAN-B*.**

**
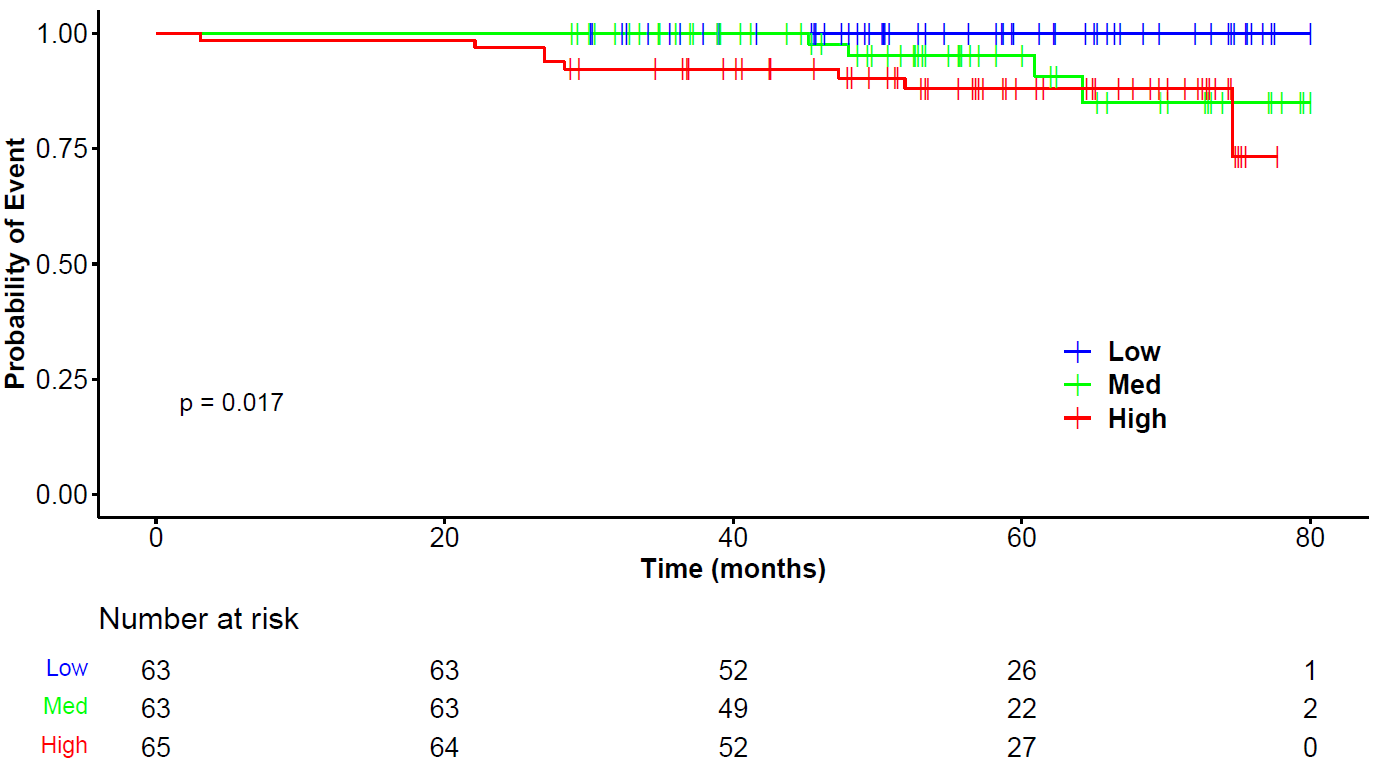
**

HER2DX pCR score was obtained from RNA-seq-based publicly available data. Tertiles were used to define low-, medium- and high-risk groups.

**Figure S13. Overall survival (OS) outcomes of HER2DX risk score according to tertiles in T1N0 and T2N0 (tumour size ≤3cm) early-stage HER2-positive breast cancer in METABRIC and SCAN-B datasets combined*.**

**
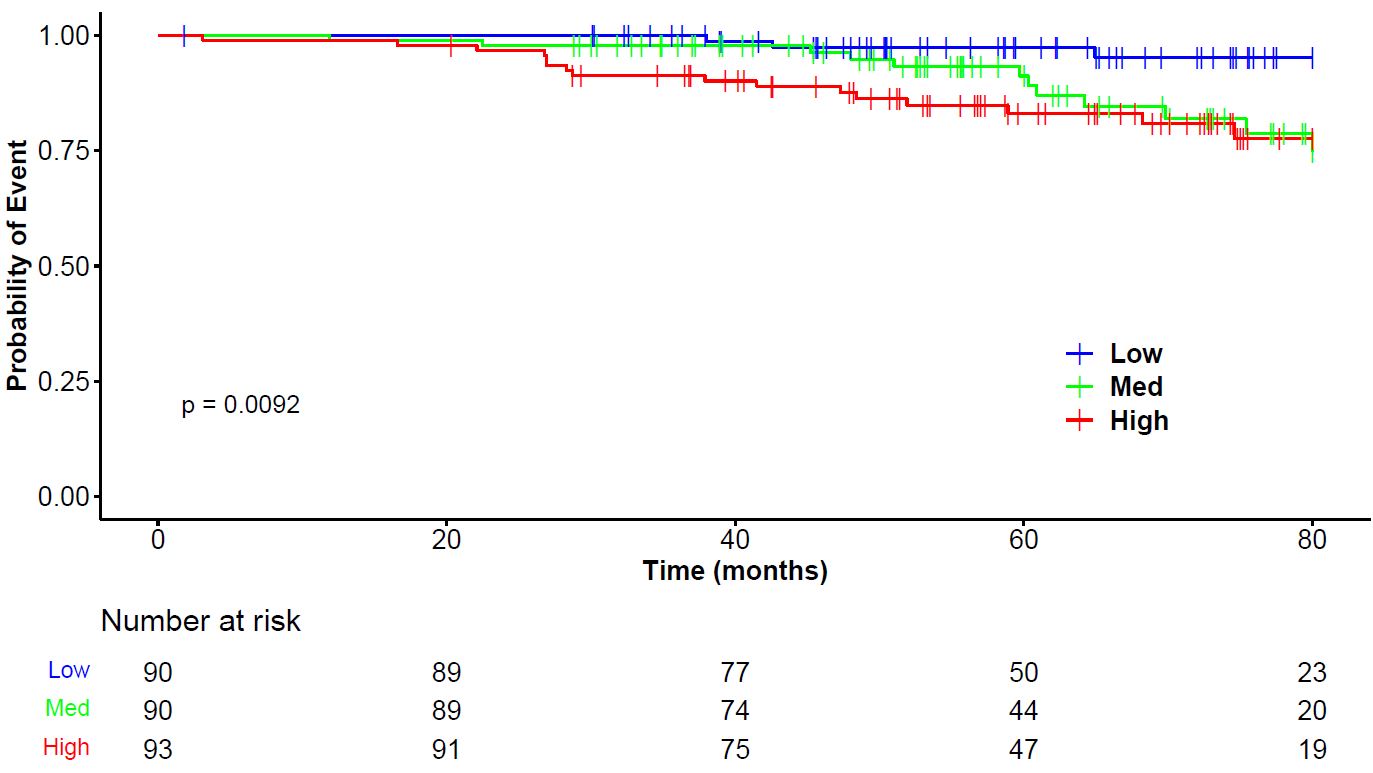
**

HER2DX pCR score was obtained from publicly available data. Tertiles were used to define low-, medium- and high-risk groups within each dataset, and then datasets were combined.

**Table S1. Publicly available genomic datasets of HER2-positive early-stage breast cancer*.**

|  | **SCAN-B** | | **TCGA** | |  | **METABRIC** | |
| --- | --- | --- | --- | --- | --- | --- | --- |
|  | **N** | **%** | **N** | **%** |  | **N** | **%** |
| **N** | 378 | - | 196 | - |  | 236 | - |
| **Age (mean)** | 59.2 | | 58.8 | |  | 56.5 | |
| **Tumour stage** |  | |  | |  |  | |
| T1 | 206 | 56.0% | 39 | 19.9% |  | 49 | 20.8% |
| T2-4 | 162 | 44.0% | 157 | 80.1% |  | 124 | 52.5% |
| Unknown | 0 | 0.0% | 0 | 0.0% |  | 63 | 26.7% |
| **Nodal stage** |  |  |  |  |  |  |  |
| N0 | 205 | 57.4% | 76 | 39.6% |  | 99 | 42.0% |
| N1 | 103 | 28.9% | 73 | 38.0% |  | 76 | 32.2% |
| N2-3 | 49 | 13.7% | 43 | 22.4% |  | 61 | 25.8% |
| **Oestrogen receptor status** |  |  |  |  |  |  |  |
| Positive | 276 | 82.9% | 152 | 77.9% |  | 104 | 44.1% |
| Negative | 57 | 17.2% | 43 | 22.1% |  | 132 | 55.9% |
| **Number of death events** | 46 | 12.2% | 24 | 12.2% |  | 147 | 62.3% |
| **Median follow-up (years)** | 4.4 | - | 2.0 | - |  | 7.2 | - |

*****SCAN-B dataset (source: GSE81540); The Cancer Genome Atlas (TCGA) dataset (source: https://www.cbioportal.org/; METABRIC dataset (source: https://www.cbioportal.org/).

**Table S2. Association of pre-treatment baseline variables with event-free survival (EFS) in CALGB-40601 publicly available dataset of 263 patients with HER2-positive early-stage breast cancer treated with neoadjuvant and adjuvant anti-HER2-based therapy*.**

|  | **Univariate** | | |  | **Multivariable** | | |
| --- | --- | --- | --- | --- | --- | --- | --- |
|  | **HR** | **95% CI** | **p-value** |  | **HR** | **95% CI** | **p-value** |
| **HER2DX risk score (continuous variable)** | 2.6 | 1.3-5.1 | 0.006 |  | 2.4 | 1.2-4.8 | 0.013 |
| **HER2DX risk groups** |  |  |  |  |  |  |  |
| High | 1.0 | - | - |  | - | - | - |
| Low | 0.5 | 0.2-0.8 | 0.012 |  | - | - | - |
| **pCR status (breast + axila)** |  |  |  |  |  |  |  |
| No | 1.0 | - | - |  | 1.0 | - | - |
| Yes | 0.5 | 0.3-0.9 | 0.030 |  | 0.6 | 0.3-1.2 | 0.180 |
| **Clinical tumour stage** |  |  |  |  |  |  |  |
| II | 1.0 | - | - |  | 1.0 | - | - |
| III | 1.6 | 0.9-2.8 | 0.150 |  | 1.8 | 1.0-3.3 | 0.052 |
| **Hormone receptor status** |  |  |  |  |  |  |  |
| Positive | 1.0 | - | - |  | 1.0 | - | - |
| Negative | 0.8 | 0.4-1.4 | 0.427 |  | 0.8 | 0.4-1.5 | 0.480 |
| **Treatment arm** |  | hg |  |  |  |  |  |
| Paclitaxel + lapatinib | 1.0 | - | - |  | 1.0 | - | - |
| Paclitaxel + trastuzumab | 0.7 | 0.4-1.4 | 0.338 |  | 0.7 | 0.3-1.3 | 0.230 |
| Paclitaxel + trastuzumab + lapatinib | 0.2 | 0.1-0.5 | <0.001 |  | 0.2 | 0.1-0.5 | <0.001 |

*HER2DX pCR score was obtained from RNA-seq-based publicly available data. Since clinical nodal status was not available, HER2DX risk score was calculated without clinical variables.

**Table S3. Association of pre-treatment baseline variables with overall survival (OS) in CALGB-40601 publicly available dataset of 263 patients with HER2-positive early-stage breast cancer treated with neoadjuvant and adjuvant anti-HER2-based therapy*.**

|  | **Univariate** | | |  | **Multivariable** | | |
| --- | --- | --- | --- | --- | --- | --- | --- |
|  | **HR** | **95% CI** | **p-value** |  | **HR** | **95% CI** | **p-value** |
| **HER2DX risk score**  **(continuous variable)** | 3.9 | 1.6-1.6 | 0.003 |  | 3.2 | 1.3-8.0 | 0.014 |
| **HER2DX risk groups** |  |  |  |  |  |  |  |
| High | 1.0 | - | - |  | - | - | - |
| Low | 0.3 | 0.1-0.8 | 0.017 |  | - | - | - |
| **pCR status (breast + axila)** |  |  |  |  |  |  |  |
| No | 1.0 | - | - |  | 1.0 | - | - |
| Yes | 0.5 | 0.3-0.9 | 0.044 |  | 0.5 | 0.2-1.3 | 0.158 |
| **Clinical tumour stage** |  |  |  |  |  |  |  |
| II | 1.0 | - | - |  | 1.0 | - | - |
| III | 2.3 | 1.0-4.9 | 0.038 |  | 2.4 | 1.1-5.2 | 0.029 |
| **Hormone receptor status** |  |  |  |  |  |  |  |
| Positive | 1.0 | - | - |  | 1.0 | - | - |
| Negative | 0.9 | 0.4-2.0 | 0.810 |  | 1.0 | 0.5-2.3 | 0.913 |
| **Treatment arm** |  |  |  |  |  |  |  |
| Paclitaxel + lapatinib | 1.0 | - | - |  | 1.0 | - | - |
| Paclitaxel + trastuzumab | 1.0 | 0.4-2.3 | 0.910 |  | 1.0 | 0.4-2.5 | 0.930 |
| Paclitaxel + trastuzumab + lapatinib | 0.33 | 0.1-1.0 | 0.052 |  | 0.4 | 0.1-1.1 | 0.085 |

*HER2DX pCR score was obtained from RNA-seq-based publicly available data. Since clinical nodal status was not available, HER2DX risk score was calculated without clinical variables.

**Table S4. Clinical-pathological characteristics of T1N0 and T2N0 (tumour size ≤3cm) across the APT trial, the METABRIC dataset and the SCAN-B dataset*.**

* P-values were obtained using chi-square tests. APT data was obtained from Tolaney et al. NEJM 2015.

| **Variables** |  | **APT** | |  | **METABRIC** | |  | **SCAN B** | |  | **METABRIC/SCAN-B** | |  | **P-value*** |
| --- | --- | --- | --- | --- | --- | --- | --- | --- | --- | --- | --- | --- | --- | --- |
|  |  | **N** | **%** |  | **N** | **%** |  | **N** | **%** |  | **N** | **%** |  |  |
| **Sample size** |  | 406 | 100.0 |  | 82 | 33.2 |  | 191 | 50.5 |  | 273 | 100.0 |  | - |
| **Age** |  |  |  |  |  |  |  |  |  |  |  |  |  |  |
| <50 |  | 132 | 32.5 |  | 20 | 24.4 |  | 57 | 29.8 |  | 77 | 28.2 |  | 0.004 |
| 50-59 |  | 137 | 33.7 |  | 24 | 29.3 |  | 51 | 26.7 |  | 75 | 27.5 |  |  |
| 60-69 |  | 96 | 23.6 |  | 23 | 28.0 |  | 45 | 23.6 |  | 68 | 24.9 |  |  |
| ≥70 |  | 41 | 10.1 |  | 15 | 18.3 |  | 38 | 19.9 |  | 53 | 19.4 |  |  |
| **HR status** |  |  |  |  |  |  |  |  |  |  |  |  |  |  |
| HR+ |  | 272 | 67.0 |  | 44 | 53.7 |  | 155 | 81.2 |  | 199 | 72.9 |  | 0.102 |
| HR- |  | 134 | 33.0 |  | 38 | 46.3 |  | 36 | 18.8 |  | 74 | 27.1 |  |  |
| **Tumour size** |  |  |  |  |  |  |  |  |  |  |  |  |  |  |
| T1mic |  | 9 | 2.2 |  | 0 | 0.0 |  | 2 | 1.0 |  | 2 | 0.7 |  | <0.001 |
| T1a |  | 68 | 16.7 |  | 5 | 6.1 |  | 6 | 3.1 |  | 11 | 4.0 |  |  |
| T1b |  | 124 | 30.5 |  | 3 | 3.7 |  | 26 | 13.6 |  | 29 | 10.6 |  |  |
| T1c |  | 169 | 41.6 |  | 45 | 54.9 |  | 100 | 52.4 |  | 145 | 53.1 |  |  |
| T2 (≤3 cm) |  | 36 | 8.9 |  | 29 | 35.4 |  | 57 | 29.8 |  | 86 | 31.5 |  |  |
| **Nodal status** |  |  |  |  |  |  |  |  |  |  |  |  |  |  |
| N0 |  | 400 | 98.5 |  | 82 | 100.0 |  | 182 | 95.3 |  | 264 | 96.7 |  | 0.114 |
| N1mic |  | 6 | 1.5 |  | 0 | 0.0 |  | 9 | 4.7 |  | 9 | 3.3 |  |  |

**Table S5. pCR rates across treatment arms in CALGB40601 neoadjuvant study according to HER2DX pCR likelihood score (tertiles)*.**

|  | **Low** | | **Medium** | |  | **High** | |  |  |
| --- | --- | --- | --- | --- | --- | --- | --- | --- | --- |
|  | **N** | **%** | **N** | **%** |  | **N** | **%** |  | **P-value*** |
| **Trastuzumab + paclitaxel (TH)** | 6/30 | 20.0% | 20/40 | 50.0% |  | 18/33 | 55.5% |  | <0.001 |
| **TH + lapatinib (THL)** | 8/32 | 25.0% | 16/36 | 44.4% |  | 28/35 | 80.0% |  | <0.001 |
| **Lapatinib + paclitaxel (TL)** | 3/16 | 18.8% | 3/19 | 16.0% |  | 10/22 | 45.5% |  | 0.067 |

*HER2DX pCR score was obtained from RNA-seq-based publicly available data (dbGAP). Since clinical nodal status was not available, HER2DX pCR score was calculated without clinical variables. P-values were obtained using chi-square tests.

**Table S6. pCR rates across treatment arms in the ISPY-2 neoadjuvant HER2-positive study according to HER2DX pCR likelihood score (tertiles)*.**

|  | **Low** | | **Medium** | |  | **High** | |  |  |
| --- | --- | --- | --- | --- | --- | --- | --- | --- | --- |
|  | **N** | **%** | **N** | **%** |  | **N** | **%** |  | **P-value*** |
| **Trastuzumab + paclitaxel (TH)** | 2/15 | 23.1% | 2/5 | 42.1% |  | 4/11 | 72.7% |  | 0.303 |
| **TH + pertuzumab (THP)** | 1/11 | 9.1% | 13/18 | 72.2% |  | 12/15 | 80.0% |  | <0.001 |
| **T-DM1 + pertuzumab (TDM1+P)** | 1/16 | 6.3% | 13/19 | 68.4% |  | 12/17 | 70.6% |  | <0.001 |

*HER2DX pCR score was obtained from microarray-based publicly available data (GSE181574). Since clinical data was not available, HER2DX pCR score was calculated without clinical variables. P-values were obtained using chi-square tests.

**Table S7. Distribution of HER2-negative and HER2-positive cases according to ERBB2 mRNA levels and pre-specified cut-off in the validation dataset.**

|  |  | **ERBB2 Low** | |  |  | **ERBB2 High** | |  |
| --- | --- | --- | --- | --- | --- | --- | --- | --- |
|  | **N** | **N** | **%** |  |  | **N** | **%** |  |
| **HER2-negative*** | 85 | 85 | 100.0% |  |  | 0 | 0.0% |  |
| **HER2-positive*** | 268 | 44 | 16.4% |  |  | 224 | 83.6% |  |

*****HER2 status defined by ASCO/CAP guidelines.
